# Technological Applications of Natural Colorants in Food Systems: A Review

**DOI:** 10.3390/foods10030634

**Published:** 2021-03-17

**Authors:** Ivan Luzardo-Ocampo, Aurea K. Ramírez-Jiménez, Jimena Yañez, Luis Mojica, Diego A. Luna-Vital

**Affiliations:** 1 Instituto de Neurobiología, Universidad Nacional Autónoma de México (UNAM), Santiago de Querétaro, QRO 76230, Mexico; ivan.8907@gmail.com; 2 Tecnologico de Monterrey, School of Engineering and Science, Avenida Eugenio Garza Sada 2501 Sur, Monterrey, N. L. 64849, Mexico; aramirezj@tec.mx (A.K.R.-J.); A00832586@itesm.mx (J.Y.); 3 Tecnología Alimentaria, Centro de Investigación y Asistencia en Tecnología y Diseño del Estado de Jalisco (CIATEJ), A. C., Camino Arenero #1227 Col. El Bajío, Zapopan, JAL 45019, Mexico; lmojica@ciatej.mx

**Keywords:** anthocyanins, betalains, carotenoids, colorants, extraction technologies, food systems, novel sources, phycobiliproteins, pigments, technological properties

## Abstract

Natural colorants have emerged as an alternative to their synthetic counterparts due to an existing health concern of these later. Moreover, natural-food colorants are a renewable option providing health benefits and interesting technological and sensory attributes to the food systems containing them. Several sources of natural colorants have been explored aiming to deliver the required wide color range demanded by consumers. This review aimed to compare and discuss the technological applications of the main natural-food colorants into food system in the last six years, giving additional information about their extraction process. Although natural colorants are promising choices to replace synthetic ones, optimization of processing conditions, research on new sources, and new formulations to ensure stability are required to equate their properties to their synthetic counterparts.

## 1. Introduction

Modification or preservation of the visible appearance of foods is perhaps one of the main applications of natural or artificial colorants [1]. Although the food ingredient industry is more devoted to develop synthetic colorants due to their stability, attractive color, and low cost, natural food-colorants are gradually being preferred due to the changing consumers’ lifestyle and increased concerns about potential adverse health effects and environmental damage caused by synthetic colorants [2]. For instance, some synthetic colorants have been linked to allergic reactions in susceptible individuals and six of them (tartrazine E102, quinoline yellow WS E104, sunset yellow FCF E110, carmoisine E122, ponceau 4R E124, and Allura red AC E129) are associated to increased hyperactive behavior in children [3]. Moreover, the use of natural colorants can provide technological and bioactive functionalities to those foods in which they are applied, delivering additional value-added properties [4].

Nowadays, natural-food colorants have found their niche for valuable food applications. Single-phase coloring systems such as baking products (solid phase) or drinks (liquid phase) have been successfully assayed with natural colorants such as carotenoids or anthocyanins (ANC) [5]. In addition, as genetical modification have been explored to increase the concentration of natural colorants in plants, there is more interest in using these procedures to increase the plants’ production yield of colorants and find more suitable applications to be used in food applications, together with technological treatments aiming to stabilize these colorants [6].

Different natural colorants have been commercially exploited and approved for their use in the USA and the European union such as ANC (grape skin extract, grape color extract, berry fruit juice, or carrot and cabbage juices), carotenoids (annatto from *Bixa orellana* L., astaxanthin from *Paracoccus carotinifaciens* or *Phaffia rhodozyma*; b-carotene from carrots, carrot oil, corn endosperm, and bell pepper from *Capsicum annuum* L.), chlorophyls from alfalfa (*Medicago sativa*), curcuminoids from turmeric (*Curcuma longa* L.), betalains from beet (*Beta vulgaris* L.) powder, carminic acid from cochineal (*Dactylopius coccus*) extract, and caramel from heating of sugars [7]. Beyond these sources, novel plants and plant-based materials and fruits, microorganisms, and insects have been considered for such purpose [8]. Colorants can be added to food systems after a technological extraction or could be part of the colored raw material. However, as some of the natural bioactive compounds that chemically constitute these colorants can be lost due to the matrix storage and processing conditions, some of them can be encapsulated to take advantage of their technological and biological properties [9]. In addition, encapsulated colorants are easier to handle and often exhibit enhanced physicochemical properties such as better solubility, stability, and flow properties [10]. The preservation of their coloring properties can be achieved by adding biopolymers such as heat-denatured whey protein isolate to reduce ANC complexation with ascorbic acid [11]. Other mechanisms involve using glutathione, dihydrolipoic acid, cysteine, and cysteine-derivatives to anchor anthocyanidins; sugars and calcium carbonate as pH-modifiers; aromatic acyl groups to acylate the 3′ position of the anthocyanin, or metal ions to form ANC-anthocyanidins complex suspended un polysaccharide matrices [12].

Food colorants play a crucial role in food production, masking unpleasant attributes or enhancing the food products’ natural properties [1]. Therefore, based on their color, they can also be used for specific purposes. For instance, ANC are highly common water-soluble flavonoids exhibiting pH-dependent colors from red to blue, and recognized by several bioactive properties such as antioxidant, anti-inflammatory, hypoglycemic, and chemopreventive effects [13]. Carotenoids are highly appreciated for their red, orange, and yellow color, primarily fruits and vegetables, contributing to desirable flavors in food and beverages [14]. Betalains are other type of colorants that have proven to be the most promissory candidates to replace Allura Red AC (Red 40), a synthetic colorant that contains benzidine, a potential human and animal carcinogen [15].

In this review, we compare and discuss some of the most recent findings in the last six years regarding the technological use of natural colorants in food systems, not only at a commercial, but also at an experimental level, providing a larger perspective on the functional aspects of colorants to be extensively used in the food industry, primarily aiming to increase the organoleptic value and enhance the natural color of food products. Furthermore, a brief description of the most important natural pigments used in the food industry and the industrial methods of production are also covered.

## 2. Natural Pigments Used in the Food Industry

**Figure 1:**
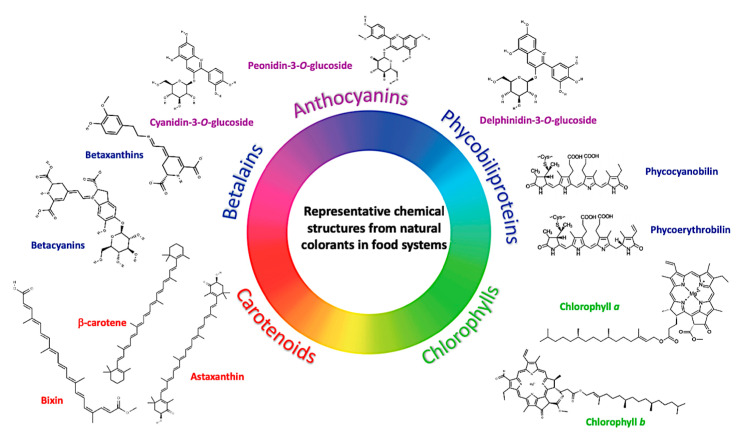
Representative chemical structures from the most common types of natural colorants applied in food systems. The chemical structures were downloaded from https://pubchem.ncbi.nlm.nih.gov (accessed on 21 February 2021). Chemical structures from phycobiliproteins were adapted from Hsieh-Lo et al. [16] with permission of Elsevier or applicable copyright owner.

Despite the wide range of natural pigments than have been used in the food industry, ANC, carotenoids, phycobiliproteins, betalains, and chlorophylls remain as the most commonly used for food applications. Some representative chemicals structures from these natural colorants are depicted in Figure 1.

### 2.1. Anthocyanins (ANC)

Anthocyanins (from Greek *anthos*, meaning flower and *kyáneos* meaning dark blue), are water-soluble vacuolar polyphenolic pigments members of the flavonoid group. Their presence in different plant organs gives the leaves, flowers, and fruits colors from red-orange to blue-purple [17]. Their basic structure is a flavan nucleus consisting of two aromatic rings: benzopyrylium and a phenolic ring joined by glucoside at carbon atom 3 of the benzopyrylium. ANC are considered the glycosylated forms of anthocyanidins since they are made up of an anthocyanidin molecule, the aglycone, to which sugar is bound through beta-glycosidic interactions such as glucose, galactose, rhamnose, and arabinose, commonly conjugated to the C3 hydroxyl group in the C-ring (Figure 1). The instability of anthocyanidins causes ANC to be found almost exclusively in their glycosylated form [17,18]. The presence of conjugated bonds in ANC results in red, blue, and purple colors, mainly depending on pH conditions [19].

What differentiates ANC from each other is the number of hydroxyl groups in the molecule, the degree of methylation of these hydroxyl groups, the nature and number of sugars bound to the molecule, their position of binding, and the nature and number of aliphatic or aromatic acids attached to the sugars [18]. The prevalent ANC forms in nature are six and represent ~90% of all ANC identified to date: pelargonidin, cyanidin, peonidin, delphinidin, petunidin, and malvidin. All of them are synthesized in plants by the phenylpropanoid pathway [17,20]. ANC are a very popular natural food-colorants susceptible to several pH-dependent color gradients, used in very popular foodstuff such as beverages, desserts, ice cream, and dairy products [12,21]. Some commercial ANC are grouped by E163 food additive, a purple colorant derived from grape skin, such as cyanidin (E163a), delphinidin (E163b), malvidin (E163c), pelargonidin (E163d), peonidin (E163e), petunidin (E163f), grape skin extract (E163ii), ANC mixture (E163ii), and blackcurrant extract (E163iii) [19,22]. Among the major health properties associated to ANC are its anti-cancer activity linked to chemopreventive and chemoprotective effects in vivo and in vitro in several cancer cell lines [23], antioxidant, and anti-inflammatory benefits [24,25].

### 2.2. Carotenoids

Based on their functional groups, carotenoids are classified in xanthophylls (oxygen-containing groups: β-cryptoxanthin, lutein, and zeaxanthin) and those having just carbon chains (α-carotene, β-carotene, and lycopene, among others) (Figure 1). Due to their hydrophobicity, they are mainly extracted using organic solvents, and depending on the natural source, the raw material might require a series of pretreatment stages [21]. Together with technological features (yellow, orange, and red color shades), most of them provide health benefits. For example, lycopene, a bioactive red colored pigment naturally found in red fruits, provides antioxidant properties with interesting health benefits linked to reduced cancer, cardiovascular disease, or diabetes risk [26]. Vitamin A is an essential carotenoid required for a plethora of metabolic purposes in the human body (immunity, growth development, and vision) [27]. Lutein and zeaxanthin provide ocular benefits and could improve the cognitive performance in elderly populations [28]. Alternative sources of carotenoids are microalgae, e.g., *Dunaliella* algae produce β-carotene under stress conditions, whereas *Haematococcus pluvialis* can produce astaxanthin [29]. Fucoxanthin is one of the most abundant carotenoids in nature, mainly extracted from brown macroalga such (class Phaeophyta: *Undaria*, *Sargassum*, *Laminaria*, *Eisenia*, *Alaria*, *Cystoseira*, and *Hijikia*), exhibiting interesting properties in those food products in which has been added, such as photoprotective, anti-obesity, anti-inflammatory, neuroprotective, anti-diabetic, antioxidant, and anti-cancer effects [30].

Some of the major technological applications of carotenoids include meat products (sausages), vegetable oils, and butter. Carotenoids are recognized as GRAS by several regulatory agencies such as the Food and Drug Administration (FDA) and the European Food Safety Authority (EFSA). However, acceptable daily intake (ADI) has been proposed for lutein (1 mg/kg body weight, BW/day), lycopene (0.5 mg/kg BW/day), zeaxanthin (0.75 mg/kg BW/day), β-carotene (<15 mg/kg BW/day), bixin (6 mg/kg BW/day), and norbixin (0.4 mg/kg BW/day) [27]. Their use as colorant additives and functional ingredients is challenging due to their water insolubility, instability, and low bioavailability, and suitable alternatives have been developed, such as carotenoid delivery in water-dispersible products, colloidal suspensions, emulsions, and colloidal dispersions [31].

### 2.3. Betalains

Betalains are water-soluble pigments chemically based on nitrogenous-functional groups (Figure 1), classified into red violet betacyanins and yellow betaxanthins. The being betanin obtained from red beetroot (*Beta vulgaris*) was the first FDA-approved betalain [21]. Betanins are used in confections, ice cream, yogurt, ready-made frostings, cake mixes, and beverages, among other applications [32]. However, betalains extracted from plants like cactus pear (*Opuntia ficus-indica* L. Mill. cv. “Gialla”: proline-betaxanthin, γ-aminobutyric acid-betaxanthin, C_15_-stereoisomers betanin/isobetanin, and 2-decarboxy-betanin)*, Celosia argentea* (miraxanthin-V or dopamine-betaxanthin, 3-methoxytyramine-betaxanthin, and (S)-tryptophan-betaxanthin), ulluco *(Ullucus tuberosus* Caldas: phyllocatin, gomphrenin III, betanin, and vulgaxanthin) [33], and *Stenocereus* sp. have been characterized [34].

Several technological efforts have been made to use betalains in food systems. However, these pigments are highly light- and high-temperature sensitive and might deliver an unappealing earthy taste to food products [8]. Nonetheless, betalains display higher water solubility, increased coloring potential, and better neutral pH-stability compared to ANC [21].

### 2.4. Other Pigments Potential Used in the Food Industry

Chlorophylls are pigments widely distributed in green fruits and vegetables, structurally composed by a porphyrin ring chelated (intramolecular bond) with a magnesium atom. Chlorophyll also contains a fifth ring beyond the four pyrrole-like rings and a chain of propionic acid esterified with phytol (C_20_H_39_) (Figure 1). The main chlorophylls found in plants are *a* and *b* in a 3:1 proportion in chloroplasts [35]. Chlorophylls exhibit several derivatives depending on high temperature, oxygen availability and changes in pH (pheophytins, chlorophyllides, phephorbides, piroderivatives, chlorin-type derivatives, and other allomerized compounds). Chlorophylls could exert biological activities such as antioxidants, antimutagenics, and anticancer activities [36]. In the food industry, chlorophylls are identified as E140i colorant and copper-chlorophylls as E141i colorant. The corresponding water-soluble forms, chlorophylins (E141ii) and copper-chlorophylins (E141ii) are also marketed. However, one of the most used chlorophyll sources is the *Spirulina* extract, which has approved use in the USA (spirulina from *Arthrospira platensis*) to be added to confections, frostings, ice cream, and frozen desserts, beverage mixtures and powders, yogurts, custards, puddings, among other food applications [37].

Phycobiliproteins are another source of blue-protein pigments, more stable compared to ANC at pH beyond the blue-range for these latter compounds (pH: 5–7) [16] (Figure 1), although ANC are more stable at acidic pH. This photosynthetic pigment is formed by fluorescent phycobiliproteins attached to the thylakoid membrane of the algae chloroplasts and chemically are built up of chromophores (bilins or open-chain tetrapyrroles) linked to thioether covalent bonds to an apoprotein [1]. Phycobiliproteins are classified into three categories: phycoerythrin (red color), phycocyanin (blue color), and allophycocyanin (bluish green color), and consist of a protein-pigment complex [38]. Phycobiliproteins can be extracted from Spirulina (*A. platensis*) phycobiliproteins, stable at pH 5.0–7.5 (25 °C) and predominantly used for color confections, gum, dairy products, and soft drinks. However, pH-stable solutions are being explored to expand their use [21]. Moreover, phycobiliproteins are very thermolabile, losing their color intensity at 60 °C for 30 min in neutral solutions. Technological approaches such as high-pressure processing have been used to pasteurize beverages at low temperatures as an alternative for using these pigments [39].

## 3. Industrial Methods of Production

### 3.1. Electric Field-Based Technologies

Electric field (EF)-based technologies are emergent processes with the potential for the rapid and uniform thermal treatment of materials. Ohmic heating (OH) and pulsed electric fields (PEF) are included in this category. Although it is not common to use these methods to extract natural colorants, some studies have addressed the potential of EF on the stability, functionality, and application of biomolecules. In addition, the electric field’s non-thermal effects (mainly electroporation) seem to enhance the extraction of compounds [40,41].

### 3.2. Ohmic Heating

With this method, an alternating electrical current is passed through a material. Consequently, the material is internally heated from the core to the outer material’s surface due to the food’s electrical resistance [42]. This feature is the main innovation of OH, making it a highly energy-efficient process suitable for rapid and uniform plant material processing. Using this technology, plants are not over-processed, and minimal changes in phytochemicals and color are produced [43].

Previous studies have used OH to extract phenolic compounds, mainly ANC, from different plant tissues [40,41,44,45,46]. The best conditions to extract polyphenols from wheat bran using OH were set at 20 V/cm, 80 °C, and 10 min, using water instead of the solvents commonly used to extract phenolic compounds [1]. A recent study also evaluated the extraction yield of ANC from *Solanum tuberosum* L. var. Vitelotte, a colored potato with blue and violet tones, using OH at different temperatures and voltages [40]. An 85% recovery of total anthocyanidins (TA) was achieved at 90 °C and 15 V after 10 min holding time, compared with a conventional thermal treatment that yielded 73% recovery. This effect depended on the time and temperature applied, but enhanced by the non-thermal effects that might cause the potato tissues’ permeabilization due to an electroporation phenomenon [40,47]. The main pigments extracted from potatoes were petunidin glucosides, malvidin, and delphinidin, responsible for the purple color. Given the antioxidant nature of ANC, this extract may be a functional colorant in foods and beverages, although its stability has not yet been studied.

A colorant powder was obtained from black rice bran (*Oryza sativa* L.) with ohmic heating-assisted solvent extraction [45]. The colorant yield obtained (up to 20.63%) was significantly higher compared to a steaming extraction process (17.64%). Dark purple ANC (cyanidin-3-O-glucoside or C3G, delphinidin, cyanidin, and pelargonidin) were successfully extracted with OH, and tocopherols such as γ-Oryzanol. Moreover, the solubility and color of the powder were not affected by the OH process; essential parameters used when evaluating the feasibility of a method for industrial applications. ANC extraction was enhanced by the electric field that causes cell wall permeabilization, allowing a higher and homogeneous release of intracellular components [41]. This effect was also observed for ANC ethanolic extraction from red grape pomace after OH application [41], with a 36% yield at 400 V/cm.

By-products such as peels and vine pruning residues (VPR) can also be used as sources of colorants. VRP is a good source of polyphenols. The OH technology has been used to obtain ethanol-water polyphenol extracts from this material (840 V/cm, 80 °C and 60 min extraction). Moreover, VP extracts may have beneficial effects on health such as antioxidant capacity, antimicrobial and anticancer activity against several cancer cell lines including HepG2, MDA-MB-231, MCF-7, and Caco-2 [48].

Color stability is an important feature when evaluating the feasibility and application of industrial systems. Processing conditions significantly affect this stability, as reported for ANC, carotenoids, and fungal pigments exposed to OH. Typically, the OH methods reach a temperature higher than 70 °C, at which phenolic compounds degrade. As shown for blueberry (*Vaccinium* spp.) pulp treated by OH, ANC degradation depends on the temperature-electric field combination [6]. At high voltages (>200 V), degradation was larger than the observed with conventional heating, and depended on the total solids content. In another study, a red extract produced by *Penicillium purpurogenum* GH2 incorporated in a beverage model system was processed with OH for microbial inactivation at 30 V and 0–80 min holding time [49]. The degradation kinetics showed lower stability for the samples treated with OH compared with conventional pasteurization. These observations indicate that the thermal effect and the concomitant influence of electric field and matrix compositions must be taken into account to maximize ANC yield and stability.

### 3.3. Pulsed Electric Fields (PEF)

This non-thermal technology uses high electric voltage in the 5–50 kV/cm range applied to food in short pulses (<1 s). Electroporation is the main accepted phenomenon occurring throughout the PEF process. Once the electric field is applied, it modifies the trans-membrane potential due to the accumulation of charges, which leads to membrane disruption and the release of intracellular components [50,51]. Since processing time is brief, degradation of bioactive compounds and natural colorants is expected to be minimal. However, several studies demonstrate that temperature, electric field strength, and time must be controlled to assure natural colorants’ optimal extraction.

Several works have extracted colorants from diverse plant species. A method based on PEF extraction of ANC was optimized for Beibinghong (*Vitis amurensis* Rupr.) [52]. The optimal conditions were found with a response surface model: four pulses at 15.08 kV to recover 166 mg ANC. In another work, PEF pretreatment for aqueous and ethanolic extraction was tested for the recovery of ANC from purple-fleshed potato (*Ipomoea batatas* L.) [53]. Processing time was the variable with the greatest effect on ANC yield, whereas electric field strength and temperature improved cell permeability. PEF allowed the use water as a solvent and decreased the temperature and processing time to obtain a similar ANC yield than the untreated samples (higher temperature and using ethanol as solvent) due to the cell permeabilization effect caused by the PEF pretreatment.

Other colorants, including betalains, astaxanthin, chlorophyll, and β-phycoerythrin, have also been extracted with PEF [54,55,56,57]. Betalains were extracted from red beet (*Beta vulgaris* L.) using PEF-assisted aqueous extraction [54]. With PEF, the maximum recovery (95%) and the minimal color degradation (10%) were reached at low temperature (30 °C), whereas the untreated samples needed higher temperature to reach >80% yield. In other study, PEF were used to disrupt microalgal biomass (*Haematococcus pluvialis*) and extract astaxanthin (pink/red color carotenoid) [55]. Applying 10–80 pulses of 5 ms at 0.2–1 kV/cm for 6 h, it was possible to observe a twofold increase in the colorant yield compared with the untreated sample. The solvent used had an important influence on the colorant recovery, methanol and ethanol were the best diluents in this experiment. In parallel work, the authors extracted β-phycoerythrin, a water-soluble red colorant present in the microalga *Porphyridium cruentum* [56]. This compound can be used in the food, cosmetic and pharmaceutical field. Interestingly, this study showed a strong correlation between the permeabilization percentage of cells treated by PEF and the extraction efficiency. Even at low intensities (~4 kV/cm), high cell damage was observed and allowed nearly a 100% recovery of β-phycoerythrin when followed by a 24 h incubation in citrate-phosphate McIlvaine buffer. These studies show the importance of testing the specific conditions to maximize cell permeabilization before colorant extraction.

PEF have shown to be a suitable method to preserve chlorophyll stability extracted from spinach (*Spinacia oleracea* L.) [14]. Once the colorant was extracted with an ethanol solution, PEF were applied to the chlorophyll extract with the following conditions: 0–26 kV/cm, 20–45 °C, and 0.32 ms as effective treatment time. The maximum chlorophyll recovery (14.62 mg/mL) was achieved at 35 °C and the highest electric field strength (26.7 kV/cm). The structural characterization by Fourier Transform Infrared (FT-IR) and X-ray (XR) diffraction showed a relatively higher stability after the PEF treatment. According to the authors, PEF induced chemical changes in the pyrrole ring and favored the formation of chlorophyll aggregates that increase the stability of the colorant. As observed, PEF can be used not only as pretreatment, but also as a method to increase or at least, to lessen colorant degradation.

### 3.4. High-Pressure-Assisted Extraction (HPE)

High-pressure-assisted extraction (HPE) obeys the isostatic principle, which states the process is volume-independent, or that pressure is transmitted instantaneously and uniformly throughout a sample, with no pressure gradients [58]. This methodology is characterized by using low or room temperatures, and pressure ranges from 100 to 600 MPa [59].

HPE is considered one of the most recent potential extraction techniques since heat is unnecessary, and therefore, the damaging effects on bioactive compounds are avoided, particularly to heat-labile compounds. As the pressure increases, there is a slight increase in temperature of 3 °C per 100 MPa, which is neglected because it is not enough to produce degradation by heat [60]. HPE has other advantages: faster extraction time from hours to minutes, lower solvent requirement, and the possibility of combining different solvents, higher extraction yields, increased extraction efficiency, and fewer impurities generation reason why it has been considered a green technology [59,60].

This methodology has become an attractive alternative for extracting bioactive compounds such as ANC since it avoids thermal degradation and oxidation reactions because of the absence of light and oxygen [61]. To mention some examples, in our workgroup, Luna-Vital et al. [62] described the ANC extraction from purple corn pericarp by using HPE. The solvent chosen for the extraction was deionized water and the process was carried out at a temperature of 50 °C for 5 min at 10.34 MPa of pressure. The extract was obtained successfully containing: C3G (45.8%), cyanidin-3-(6′-malonylglucoside) (C3G-Mal) (17.2%), a condensed form of flavanol-ANC (16.8%), peonidin-3-O-glucoside (P3G) (9.3%), peonidin-3-(6′-malonylglucoside) (P3G-Mal) (3.1%), pelargonidin-3-(6′-malonylglucoside) (Pr3G-Mal) (2.4%), and pelargonidin-3-*O*-glucoside (Pr3G) (2.0%). However, ANC were not the unique components of this extract since several phenolic acids were also extracted, such as ferulic, protocatechuic, caffeic, chlorogenic, and gallic acids [63]. Putnik et al. [64] evaluated the performance of the high hydrostatic pressure extraction (HHPE) on the recovery of ANC from the grape skin pomace extracts under moderate temperatures. In this case, two solvents were used (ethanol and methanol), the compression fluid was propylene glycol, and the conditions of the extraction process were the following: 300, 400, and 500 MPa of pressure for 3, 6.5, and 10 min at 22, 26, and 30 °C. The authors obtained, mainly, malvidin derivatives in two forms: malvidin-3-glucoside (2.33 mg/g) and malvidin-3-*O*-(6-*O*-acetyl)-glucoside (0.83 mg/g), representing 55.77% of overall ANC content. Followed by malvidin-3-*O*-(6-*O*-coumaroyl)-glucoside (0.66 mg/g) being 11.65%, the remaining ANC were found in amounts lower than 0.5 mg. Once analyzed the applied parameters, the ideal settings for the process were pressure 268.44 MPa, extraction time 3.39 min, and temperature 29.48 °C.

Haining and Yongkun [65] had the purpose of evaluating the influence of HHPE on the extraction of blueberry pomace ANC. The pressurizing fluid used was dioctyl sebacate, and the chosen solvents were ethanol and hydrochloric acid. The extraction process was carried out under different pressures (100 and 600 MPa) and holding times (5 and 30 min) at room temperature. According to their optimization model, the significant extraction parameters were a liquid-solid ratio of 41 mL/g, ethanol concentration of 63%, and extraction pressure of 443 MPa. The non-significant parameters such as hydrochloric acid concentration, holding time, and extraction cycles were fixed at 0.185%, 5 min, and 1 cycle, respectively. At the optimal HHPE conditions, 107.9 mg/100 g of ANC were obtained, and 10 ANC were identified, being malvidin-3-galactoside and malvidin-3-glucoside the main ones.

### 3.5. Supercritical Fluid Extraction (SFE)

Supercritical fluid extraction (SFE) is a process used for separating one component, named the extractant, from another known as the matrix, using supercritical fluids as the extracting solvent [66]. The conditions of these fluids are above their critical point of pressure and temperature, their density is similar to liquids, their viscosity is comparable to gases, and their diffusivity is between both gases and liquids [67]. The main advantage of a supercritical fluid is that its density can be modified by changing its pressure and temperature. The properties mentioned earlier allow supercritical fluids to penetrate deeper and faster to solid matrices because they diffuse easily through them [67,68].

Carbon dioxide (CO_2_) is the most commonly used solvent due to its low cost, safety, and moderate critical temperature (31.2 °C) that enables the preservation of bioactive compounds in extracts [67,69].

Other remarkable advantages in comparison with standard extraction techniques are the use of solvents generally recognized as safe (GRAS), lower extraction times, increased yields meaning higher efficiency of the extraction process, and the option of direct coupling with analytical chromatographic techniques such as gas chromatography (GC) or supercritical fluid chromatography (SFC) [68]. Jiao and Kermanshahi [70] obtained ANC extracts from Haskap berry (*Lonicera caerulea*) pulp by SFE. Likewise, the authors carried out the extraction of ANC by the conventional method using water, intending to compare the yields obtained. A relevant aspect of this work was the combination of water and CO_2_. The highest total ANC yield (52.7%) from berry pulp paste using CO_2_ was achieved using 45 MPa, 65 °C, 5.4 g water to 3.2 g paste, 15 min static, and 20 min dynamic time. In conclusion, compared with conventional extraction, using CO_2_ as solvent and water use as co-solvent offered higher ANC extraction efficiency (52.7% vs. 38.3%).

The recent work conducted by Idham et al. [71] aimed to evaluate the effects of different particle sizes, flow rates, and modified ratios on the extraction yield and ANC content of Roselle (*Hibiscus sabdariffa*) by using the supercritical carbon dioxide (SC-CO_2_) method. The pressure and temperature were kept constant at 10 MPa and 70 °C respectively, and 75% ethanol was used as a modifier. Different solvent flow rates were studied: 4 mL/min, 5 mL/min, and 6 mL/min. Three different ground dried Roselle sizes were used: 200–355 μm, 355–500 μm, and 500–710 μm. Finally, three percentages of modifiers ratios were compared: 5%, 7.5%, and 10%. The effect of these three parameters showed different results of overall extraction yield and total anthocyanin content (TAC), demonstrating that these conditions are key to obtain the highest ANC concentration. The optimal parameters that allowed reaching the highest ANC concentration were extraction time of 120 min, flow rate of 4 mL/min obtaining a TAC of 5 mg equivalents of C3G (EC3G)/L, particle size of 200–355 μm showing a TAC of 4.95 × 10^4^ mg EC3G/L, and a 10% of modifier ratio obtaining a TAC of 3.84 × 10^4^ mg EC3G/L. A summary of the primary outcomes from reported industrial extraction technologies of natural colorants is presented in Table 1.

## 4. Technological Properties of Natural Food Colorants in Food Systems

**Figure 2:**
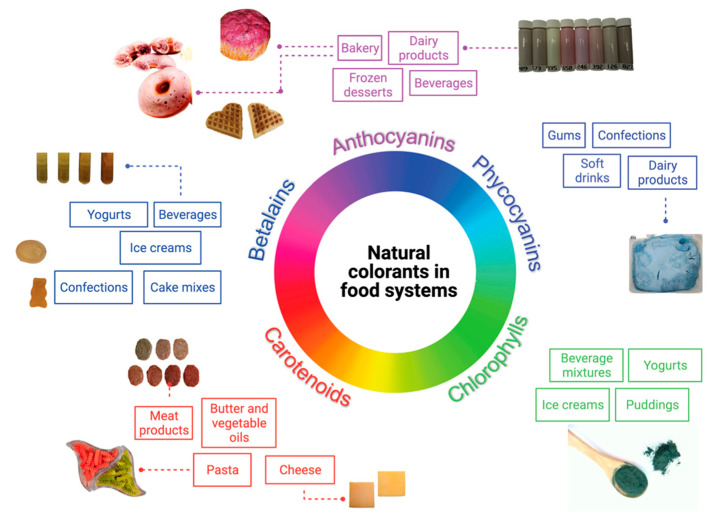
Natural colorants in food systems. Figures reprinted from Abdel-Moemin et al. [73], Amjadi et al. [74], Carballo et al. [75], da Silva et al. [76], de Amarante et al. [77], Freitas-Sá et al. [78], Jiménez-López et al. [79], Rodríguez-Sánchez et al. [34], Sharma et al. [80] with permission of Elsevier, MDPI A. G., or applicable society copyright owner.

Several food colorants have been isolated from diverse sources to be applied in food systems. An overview of some of the most important food systems in which natural colorants are applied is presented in Figure 2.

### 4.1. Bakery Products

Purified colorants or plant extracts have been used to improve bakery products’ nutritional properties without a major sensory impact. As such, Abdel-Moemin et al. [73] reported an enhanced chemical composition and high overall liking scores from cupcakes added with 20 g Roselle (*Hibiscus sabdariffa* L.) calyces extract/100 g cupcake, commonly used in the preparation of beverages. This functional extract was prepared with dry calyces to produce a fine powder (0.55 mm) mixed with water and then boiled for 1 h (80 °C). The formulated cupcakes showed lower total carbohydrates (−11.28%) and lipids (−16.48%), while higher dietary fiber (126.18%) and ash (179.68%) than the control cupcakes (without Roselle). Moreover, Roselle-added cupcakes retained 77% of the total ANC content from the dry calyces (435 mg/100 g cyanidin-3-glucoside), potentially providing up to 32-fold the minimum daily intake from Americans (12.5–215 mg). Although the resulting cupcakes displayed a crust and crumb pink color due to surface Maillard reactions developed during the baking process (175 °C for 20 min), no differences (*p* > 0.05) were found for the sensory evaluation of the color, appearance, texture, taste, volume, and aroma compared to control cupcakes, but received a lower liking.

Jiménez-López et al. [79] assessed the feasibility of a C3G extract from *Arbutus unedo* L. fruits to be incorporated into wafers. An optimized heat-assisted extraction was used to obtain an antioxidant C3G-rich extract with antioxidant potential (2,2-diphenyl-1-picrylhydrazyl or DPPH half-maximal effective concentration, EC_50_: 295 μg/mL and β-carotene EC_50_: 901 μg/mL) with anti-bacterial properties (*Salmonella enteritidis* minimum inhibitory concentration (MIC): 150 μg/mL; *Salmonella typhimurium* minimum bactericidal concentration (MBC): 200 μg/mL). The extract exhibited the highest stability at T < 20 °C and pH > 3.5. When added to wafers, the prepared product showed a golden color, higher sucrose amount, increased concentrations of fatty acids (palmitic, stearic, and linoleic acids), and improved antioxidant capacity compared to the untreated wafers.

Fruit and vegetable waste can also be a source of colorants. Tomato waste was employed as a lycopene source to be used in cakes and cookies [26]. Lycopene from tomato waste (fibrous pulp without peel and seeds) containing 654.8 mg/100 total carotenoids and 300.85 mg/100 g lycopene. Oil from the cake’s formula and butter from the cookies’ ingredients was replaced with 1%, 3%, and 5% lycopene, and the resulting products were evaluated. Lycopene-containing cakes showed a dose-dependent volume increase, higher DPPH inhibition, and increased crust and crumb’ lightness, but only 5% formulation showed a higher volume than control cakes (without lycopene). Sensory evaluation of the cakes showed significant differences in crust and crumb’s color and texture (cakes were perceived as more yellow and redness than control cakes). Still, no differences were found among panelists for taste, odor, and overall acceptability. The same outcomes were found for the lycopene-added cookies.

Beetroot (*Beta vulgaris* L.) pomace was used as a source of betacyanin and betacyanin-derivatives extract, further encapsulated and used in pseudocereals (amaranth, buckwheat, and quinoa)-enriched wheat einkorn (*Triticum monococcum*) water biscuits. Independently of the pseudo-cereal, all extract-added biscuits showed a dose-dependent betanin, isobetanin, and betanin-derivatives increase (5.7%, 10.4%, 14.9%, and 10.8% extract addition) compared to control biscuits. Buckwheat (*Fagopyrum esculentum*) biscuits displayed the highest total phenolic compounds (TPC) content (~2500 mg gallic acid equivalents or GAE/kg dry matter, DM), ferric ion reducing antioxidant power (FRAP), and 2,2′-azino-(bis-ethylbenzothiazoline-6-sulfonic acid) (ABTS) values (~18 and 14 mmol Trolox equivalents/kg DM, respectively). Quinoa (*Chenopodium quinoa*) biscuits showed the highest furosine contents (~275 mg/100 g protein), suggesting a lower ability of the encapsulated extract to prevent heat damage for this ingredient.

*Rubus ulmifolius* Schott has been investigated as a novel source of food colorants incorporated in bakery products [76]. Heat-assisted extraction was conducted to produce an ANC-rich extract, and the main identified ANC (cyanidin-*O*-di-hexoside, C3G, Pr3G, cyanidin-3-*O*-xyloside, and cyanidin-3-*O*-dioxayl-glucoside) were used as responses for a Response Surface Analysis (RSM). The extract contained 33 mg ANC/g extract and showed a red-burgundy color. When added to donuts, lightness and yellowness (b*) decreased (−24.34 to 25.97% and −44.67 to 48%, respectively), but the redness was increased (+109 to 338.67%) when compared to control donuts. The formulated donuts also showed lower carbohydrates and energy value contents, higher free sugars values (*p* < 0.01), and no differences were found for the free fatty acids content.

Albuquerque et al. [81] optimized a heat- and ultrasound-assisted ANC-rich extract from jabuticaba (*Myrciaria jaboticaba* (Vell.) Berg) epicarp as a natural colorant to be used in the manufacturing of french macarons. Heat-assisted extraction proved to be the most successful extraction using delphinidin-3-*O*-glucoside and cyanidin-3-*O*-glucoside levels as responses. The resulting macarons (13 min at 130 °C, conventional oven) showed lightness (L*), redness (a*), and yellowness (b*) preservation (overall −0.05% change) up to six days of storage, while low glucose, fructose, and sucrose changes were observed during the same evaluation period.

Several reports have informed the potential of whole food products for coloring or technological properties. For instance, colored tubers such as purple-fleshed sweet potato (*I. batatas* L.)-colored flours were used in biscuit formulations [82]. Although no colorants were mainly extracted from the raw materials, the flour contained high TPC (80.89 mg GAE/100 g) and TAC (38.90 mg C3G equivalents/100 g). Despite 67.24% TPC and 27.79% TAC were retained in the biscuits, respectively, due to compound losses during the baking process (160 °C, 20 min, electric oven), potato-supplemented biscuits exhibited TPC: 2.27- and TAC: 10.82-fold increase, compared to control biscuits. The enhanced nutritional composition yielded high FRAP and DPPH levels.

Similarly, Croitoru et al. [83] partially replaced wheat flour with black rice flour to manufacture muffins. The 50% and 100% black rice formulations showed an outstanding TPC, total flavonoids (TF), and TAC contents, compared to wheat-only muffins, which was reflected on the antioxidant capacity (up to four-times compared to control muffins). No differences were found (*p* > 0.05) for the overall acceptability of the novel muffins, showing a beneficial potential of colored ingredients to improve the nutritional composition without a negative impact on the sensory parameters.

In summary, natural food-colorants can be used to positively impact the crust and crumb’s color from several bakery products, producing a pleasant flavor and interesting health-added benefits, mainly antioxidant properties. However, most researchers have not evaluated these properties at in vivo level but in vitro tests using assays that hardly mimics the antioxidant levels found in organisms [84]. Moreover, more research is needed evaluating the impact of the processing conditions at which bakery products are subjected (high temperature, low moisture, among others) in the dyeing properties of natural food-colorants. Hence, encapsulation might be a suitable technological advantage using proper wall materials allowing colorants to exert their function but also preserving their healthy characteristics.

### 4.2. Beverages

Consumers usually associate the beverages’ colors from natural sources such as yellow for lemons or red for strawberries, to mention some examples. Hence, color is a critical feature with the potential to enhance their appeal and acceptability [85].

Most beverages require colorants since food processing contributes to substantial color loss. Thus, various food and non-food natural sources can be used as materials to isolate functional colorants, retaining stability and shelf life. Commercial purple carrot ANC (0.025%) combined with green tea extract rich in epigallocatechin gallate (EGCG) showed high stability to ANC degradation and delayed half-life of ANC from 2.62 days to 6.73 days in first-order reaction kinetics [13]. These protective effects provided by the green tea extract are a consequence of EGCG protection over ascorbic acid condensation or oxidation by hydrogen peroxide, allowing even higher stability at increasing temperatures (from 25 °C to 40 °C).

Cyclodextrins are cyclic molecules used as encapsulants of flavors, vitamins, colorants, and ingredients, increasing their shelf-life and promoting controlled release for technological or physiological features such as improved bioaccessibility, and antioxidant capacity [86]. Particularly for beverages, it can be used for co-pigmentation purposes, stabilizing highly degradable pigments. For example, black bean coats anthocyanin-rich extracts were stabilized with 2% β-cyclodextrins to manufacture a model sport beverage [87]. The extracts were prepared using coats from two black beans (*Phaseolus vulgaris* L.) varieties (“Negro Otomi” and “Idaho” cultivars) after an optimized pH-adjusted aqueous ANC extraction (40 °C for 4 h). High destruction values were obtained for the ANC powders and coat extracts (9.51–119.93 days), but encapsulation with β-cyclodextrin retarded their degradation (up to 43 months). β-Cyclodextrin widely used as additive to protect colorants from environmental conditions. The addition of the β-cyclodextrin-encapsulated extracts to a sports beverage extended ANC half-life (up to 13 months) and reduced color differences under darkness at 4 °C.

Beverage models can be used to evaluate the stability of natural colorants at varying pH, acidity, and other environmental conditions. Rodríguez-Sánchez et al. [34] evaluated pitaya (*Stenocereus pruinosus*) betaxanthins (betalain-type pigments) extracts in a model yellow beverage, obtaining a highly disperse chroma (21.38–87.78) and hue (53.9–87.8) values and several shades of yellow-orange. However, beverages pigmented with 5% pitaya juice were those with the most similar color to their commercial counterparts (color difference ΔE = 9 vs. control beverage). Formulated beverages retained up to 75% of total betaxanthins during the first nine days of storage. The authors suggested the ability of this pigment to substitute synthetic yellow pigments in commercial beverages.

Lobo et al. [88] encapsulated yellow bell pepper pigments with β-cyclodextrin and evaluated their stability in isotonic beverages (pH: 2.9; 0.02%, 0.05%, and 0.06% of extract addition). Lutein, zeaxanthin, α-cryptoxanthin, α-carotene, and β-carotene were the main carotenoids found. Extract-added beverages exhibited dose-dependent luminosity and redness increase but decreasing yellowness. No differences along time (21 days) were observed among the beverages for lightness, but yellowness significantly decreased (*p* < 0.05).

Soft-drink beverage models were assayed to evaluate the stability of a vacuum-concentrated colorant extracted from yellow-orange cactus (*Opuntia ficus indica*) [89]. The extract (0.7% *v/v*) was added to soft drinks (86 g/L sucrose, 0.14 g/L sodium benzoate, 0.18 g/L potassium benzoate, 0.02 g/L ascorbic acid, and 1.52 g/L citric acid, among other components). Indicaxanthin was the main identified betaxanthin (concentration: 256.53–264.86 mg indicaxanthin equivalents/kg). First-order reactions were fitted for betaxanthin degradation in the soft-beverage models, where the beverages’ pH (3.0) could influence the degradation as betaxanthin are stable at pH: 5.0. As suggested by the authors, this extract could also be used in chilled matrices such as yogurt and ice cream.

The yellow color is a very demanded pigment in the beverage industry, being carotenoids (bixin, lutein, and crocin), betalains (betaxanthins), flavonoids (carthamin), curcuminoids (curcumin), and riboflavins the most usual colorants [90]. An extensive evaluation of several yellow natural pigments (annatto from *Bixa orellana* L. seeds, gardenia yellow from *Gardenia jasminoides* Ellis, lutein from marigold flowers or *Tagetes erecta* L., curcumin from turmeric or *Curcuma longa* L., and safflower extract from *Carthamus tinctorius* L.) were evaluated in colored beverage model systems [91]. To simulate alcoholic and non-alcoholic beverages, McIlvaine buffer (pH: 3.5, 5.5, and 7.5; concentrations: 0.001, 0.005, 0.01, 0.02, 0.03, 0.05, 0.10, and 0.30% *v/v*) with or without ethanol (15% *v/v*), were prepared. Gardenia, safflower, and curcumin exhibited the highest color intensity and the lowest turbidity level, whereas safflower showed the highest heat (25, 40, 60, and 80 °C) and light (550 W/m^2^, 30 °C) stability.

Cyanidin-rich or pelargonidin-rich purple corn extract-added model beverages were tested for stability with and without a flavone-rich extract [92]. The cyanidin-rich beverages were more stable than pelargonidin ones, but a 50% increased half-life was obtained for both systems after adding the flavone extract. Together with their technological features, these extracts have also shown interesting biological properties in vitro and in vivo, playing a dual role in replacing artificial colorants and delivering potential health benefits [63]. Compared to extract-added-only beverages, the addition of alginate and zinc ions to these extracts protected ANC from degradation in a beverage model, improving ANC half-life (10.4 weeks), C3G concentration (7.5 weeks), and chroma (18.4 weeks) [62].

Gomes Rocha et al. [93] developed whey-based beverages containing ANC from jabuticaba (*Plinia cauliflora*) skins for protein beverages. Delphinidin-3-*O*-glucoside (D3G: 9.8 mg/L) and C3G (198.9 mg/L) were identified in the jabuticaba extract. The highest whey-containing beverages (4.0% and 6.0% *w/v*) exhibited clearer colors, with no differences in a* and chroma parameters. The red color was predominant in the beverages. All beverages contained the same (*p* > 0.05) ANC content (1.4–1.5 mg/100 g), but TPC and antioxidant capacity were whey dose dependent.

Jelly drinks were pigmented with encapsulated ANC pigments from purple sweet potato (*Ipomoea batatas* L.) cv. Ayamurasaki [94]. The resulting beverage stored at 5 °C without light exposure showed the smallest ANC and redness decreased after 30 days of storage, while its calculated shelf-life was estimated in 200 days.

Among alga-derived sources, *Porphyridium aerugineum* microalga-derived blue color has been used for acidic beverages. These pigments display pinkish-red or blue color extracted by cell breakage using water or buffered solutions, centrifugated, purified, and sterilized by microfiltration, spray-drying, or freeze-drying [95]. The blue color is stable at pH 4.0–5.0 for 1 month at room temperature or up to 40 min at 60 °C. It can be added to acidic non-heat treated carbonated beverages (Pepsi ^®®^) or low-grade alcohol beverages (Bacardi Breezer ^®®^) [1,95].

Concluding, beverages are one of the most suitable models for studying the natural food-colorants’ shelf life since the aqueous media and processing conditions provide challenging conditions to test colorants’ behavior. Since beverages have been used to promote the consumption of vegetables and fruits as their intake remain below the recommended levels in many countries [96], colorants are also used to prevent beverages’ color loss during the thermal processing.

### 4.3. Confectionery

Confections belong to a very dynamic food industry sector with high demand for coloring agents. Several confections have been targeted as concerning due to cadmium and lead-based colors potentially causing brain glioma, urinary bladder and kidney tumors, or hypersensitivity [97].

Figs (*Ficus carica*) and blackthorns (*Prunus spinosa* L.) extracts were used to manufacture *beijinho*, a condensed milk-based confection, and doughnut icing [98]. Peels from *F. carica* and the epicarp of *P. spinosa* were freeze-dried, milled (20 mesh), and ultrasound-assisted extraction was conducted, using 100 mL of acidified solvent (pH 3, citric acid). The peel was rich in cyanidin-3-rutinoside, while the blackthorns extracts were rich in cyanidin-3-rutinoside and peonidin-3-rutinoside. Both food products contained sucrose > lactose > fructose contents. Up to 21 fatty acids were identified in both foods, and palmitic acid was the most abundant (icing: 14%; *beijinhos:* 10%). Saturated fatty acids were the most relevant group (75%), followed by monounsaturated. Blackthorn-added products displayed intense purple colors, and figs-added products exhibited light tones.

A betaxanthin-rich extract from pitaya (*S. pruinosus*) was used to produce yellow gummies, showing disperse chroma values depending on the juice or pulp concentration (10.6–15.6 and 10.6–13.2, respectively), with shading varying from yellow to orange. Half of the pigment was lost after 11 days at 40 °C, and betaxanthins followed a first-order kinetic evolution [34]. Calcium alginate-encapsulated betalains from *Opuntia ficus-indica* (purple pulp) were added to gelatin-based gummies, and no differences in the color parameters (lightness, a*, and b*) were found after a 30 days-storage at 4 °C [99].

Saffron (*Cocus sativus*) and beetroot (*Beta vulgaris* L.) aqueous extracts were encapsulated in maltodextrin, gum arabic, modified starch, and chitosan and incorporated in a chewing gum system [100]. Modified starch and other ingredients were used as wall materials to protect target pigments in food systems [101]. The luminosity from the chew gums decreased along time (25 °C and 40 °C storage), whereas a* almost disappeared after two-weeks’ storage at 40 °C. However, gum arabic and modified starch proved to be the best color stability mixture independently of the extracts, displaying the highest a* (for beetroot) and b* (for saffron) values.

Promising underutilized fruit products can be used as sources of varied colorants for the confection industry, despite a colorant not being extracted from these raw materials. Açaí (*Euterpe oleracea* Mart.) was evaluated for its coloring properties in a chewy candy model [102]. The resulting candies had lower a_w_ and no differences were found for the hardness or the moisture content than non-added Açaí candies. Higher color acceptance (4.34%) and the same texture evaluation were obtained for both non- and added-Açaí candies. Although the positive purchase intent was lower in the Açaí candies (−42.86%), high uncertainty in the purchase intent (166.7%) was observed in the Açaí testers, suggesting the potential of these candies to be acquired.

Dragees or hard-panning confections are elaborated by applying several layers of coating material such as saturated sugar syrup to produce a hard or crispy shell [103]. Most water-soluble pigments can be used in these confections, but as the panning syrup is prepared at temperatures higher than 80 °C to prevent recrystallization of sugar, the tested pigments require to preserve most of its properties at these working conditions [104]. Avelar et al. [105] explored the possibility of using by-products from Uvaia (*Eugenia pyriformis*), a native fruit from southeastern Brazil, as a low-cost coloring agent in hard panning confections. Coated confections exhibited the lowest a_w_ and luminosity, but the highest a*, b*, and hardness than fruit-based concentrate- and artificial colorant-added candies. The best appearance and color attributes were obtained for the Uvaia-added candies, but they displayed the lowest crispness (6.61 score vs. 6.77 and 7.16 for fruit-based and artificial colorants).

Several pigments can be used to color jelly gummies, confections composed of sugars, gelling agents (pectin, agar-agar, gum arabic, gelatin, among others) and food-grade acids (citric or tartaric acids). For the yellow shades, Carthamus (*Carthamus tinctorius* L.) has been used to provide a transparent appearance, but bright yellow can be reached using curcumin. However, these oily pigments need to be formulated using proper emulsion, suspensions, or encapsulation systems since curcumin exhibits poor stability [104]. For warmer color shades, carotenes can be applied to jelly gum from varied sources such as palm carotenes, resulting from the fungal fermentation of *Blakeslea trispora*, or those produced by the alga *Dunaliella salina.* Nonetheless, for another color, unconventional or underutilized fruit and vegetable sources can be used. Małgorzata et al. [106] reported the potential of black elderberry (*Sambucus nigra* L.) extracts prepared from their flowers and fruits as dyes to manufacture healthy jelly gum confections. ANC such as cyanidin-3-*O*-sambubioside-5-glucoside, cyanidin-3,5-diglucoside, cyanidin-3-*O*-sambubioside, and cyanidin-3-*O*-rutinoside were identified in the extracts. However, the flowers exhibit a rich polyphenolic profile governed by quercetin derivatives (4.04 mg/g quercetin-3-rutinoside and 0.56 mg/g quercetin-3-glucoside) and chlorogenic acid (2.82 mg/g). The designed jelly gummies showed high FRAP (597.46–849.58 μmol Trolox equivalents, TE/g), DPPH (68.23–90.11% inhibition), and TPC (14.68–25.34 mg GAE/g).

Microalgae are an important source of natural pigments, containing macro and micronutrients with interesting biological properties that could add health benefits to natural colorants in confections [107]. For example, Genc Polat et al. [108] applied spray-dried encapsulated *Nannochloropsis oculata* microalga extract in white chocolate as a coloring agent. The obtained chocolate presented varied luminosity (61.6–78.0) and increased a* and hue compared to control chocolate samples. Although the alga-added samples showed lower scores for the appearance, texture, and smell, their values were not different (*p* < 0.05) than the control samples.

The red microalga genus *Porphyridium* has proven to be a source of fluorescent phycobiliproteins with pigment properties on confections. The red or pink colorant can be added to transparent lollipops made from sugar solutions or dry sugar-drop candies for cake decorations, exhibiting high stability at 60 °C for 30 min and long shelf life at pH 6.0–7.0 [1].

Last but not least, adding natural food-colorants to confection opens an opportunity to diversify the functional confectionery market, reaching traditional population targets such as children, to deliver health benefits in low sugar formulations with increased nutritional properties. Functional confectionery no only relies on adding isolated natural food-colorants, but also food by-products with demonstrated beneficial nutritional composition such as high dietary fiber and antioxidant compounds contents [109].

### 4.4. Milk, Dairy, and Dairy-Like Products

Similar to other food products, natural food-colorants can be added to milk and dairy products to restore the natural color potentially lost during processing and storage or to reduce the batch-to-batch variations. Moreover, colorants can intensify natural colors in case they are weak, provide color to colorless products, and produce acceptable and attractive products for consumers [8]. Several researchers have used natural colorants in milk and dairy products for several purposes, such as those described above.

Dairy food matrices are challenging since colorants might affect some of their textural properties. Natural colors are usually preferred for yogurts due to their heat stability during processing and can be easily labeled as “vegetable color”. Some of the most common natural colorants used in yogurts are carmine from cochineal beetle (brilliant red color ranging from “strawberry” to “blackcurrant” color), annatto from *Bixa orellana*, ANC-rich extracts from mulberry (*Morus rubra*), red color from strawberries, and orange color from the carrot addition to yogurt [110]. Betacyanin pigments extracted from Ayrampo (*Opuntia soehrensii* Britton and Rose) were used in 3% fat yogurt as natural colorants. After an optimized extraction and purification, betacyanins showed first-order degradation kinetics when subjected to heat treatment (80 °C for 90 min) at several pHs (3, 4, and 5) [111]. However, an average half-life of 272 days was obtained when betacyanins were stored at 4 °C, whereas a half-life of 26 days was shown for 25 °C storage. The application of this extract in yogurt (32–192 μg betacyanin/100 g yogurt) resulted in the lowest ΔE at 96 μg betacyanin/100 g, while greater values exhibited decreased L* values and higher ΔE, compared to commercial yogurt. Color stability during storage (up to 5 weeks) was similar to synthetic colorant Red no. 40 (>94%), and even higher than red beet extracts also applied to the same yogurts.

For example, natural curcumin (E100) was applied to a hydrophilic matrix represented by yogurt [112]. As curcumin exhibits poor water solubility and susceptibility to alkaline conditions, light, oxidation, and heat, encapsulation was also assayed. Diverse color changes (closer to organ color) were obtained from the formulations (4.75–5.25%), where water, protein, ash, galactose, energy, and b* were the dominant parameters having a discriminant effect among the samples. Stored yogurts for seven days displayed a* reduction, but overall color could be maintained a long time.

Pires et al. [113] incorporated natural colorants extracted from flowers such as *Dahlia mignon*, *Rosa damascena* “Alexandria”, *Rosa gallica* “Francesca”, and *Centaurea cyanus* L. *Centaurea* contained the highest amount of TAC (26 μg/g), and cyanidin-3,5-di-*O*-glucoside and cyanidin-3-*O*-(6′’-malonylglucoside)-5-*O*-glucoside were the most abundant ANC. Except for the galactose contents (*Centaurea* was the highest), there were no differences (*p* > 0.05) for the water, fat, protein, ash, carbohydrates, lactose, or energy contents and these parameters did not change during the seven-day storage of the yogurts. Furthermore, compared to a commercial colorant (E163), there were no differences in L*, a*, b*, or pH values.

Jabuticaba (*Myrciaria jaboticaba* (Vell) O. Berg) and *jamelão* (*Syzygium cumini* L. Skeels) peel powders were testes as colorants in yogurts [78]. Test consumers (106) participated in a matching task associating the manufactured yogurts with fruit flavor variants, and high dependence (*p* < 0.01) between color and flavors of yogurts was found. For the sensory evaluation, Jabuticaba-added yogurts received the best appearance (6.6–6.8), flavor (6.9), and overall liking (6.8) scores among the flavored-yogurts. However, all values were lower (*p* < 0.05) than the not-colored yogurt except for the appearance.

Benchikh et al. [114] optimized ANC extraction from strawberries (*Fragaria ananassa*) using the response surface methodology and applied the obtained colorant to yogurt. ANC’s optimal conditions were obtained for agitation speed of 586 rpm, and sample to solvent ratio of 1.26 g/40 mL, obtaining TAC: 38.04 mg C3G equivalents/100 g fresh weight, and 21.22 μg ascorbic acid equivalents/100 g yogurt for the antioxidant capacity (DPPH). The yogurts contained 10–40 μg/100 g TAC and a remaining red color after the manufacturing process (pH: 4.6, 4 °C), but no shelf-life evaluations were conducted.

The color stability of betalain- and ANC-rich extracts in yogurt-like fermented soy from several sources such as red beetroot (*Beta vulgaris* L.), opuntia (*Opuntia stricta*), Roselle (*Hibiscus sabdariffa*), and radish (*Raphanus sativus* L.) was evaluated [115]. The authors showed that red beetroot contained the highest amount of betacyanins (20.1 mg equivalents of betacyanin/L) and betaxanthins (4.27 mg equivalents of indicaxanthin/L), while Roselle the highest quantity of ANC (13.01 g/L). The extracts were applied in an encapsulated and non-encapsulated form (liposomes of soybean lecithin), showing high retention of the extracts after 21 storage days, being Roselle and red radish, the most stable ones compared to untreated yogurt samples. The beetroot-added yogurts exhibited the highest a* values, followed by red radish, opuntia, and Roselle.

Flavored fermented milk was prepared with a microencapsulated extracted pigment (Canthaxanthin), a carotenoid-type colorant from *Dietzia natronolimnaea* HS-1 bacteria, which is permitted to be used in milk products up to 15 mg/L [116]. Fermented milk beverages showed significant reductions (*p* < 0.05) in the antioxidant capacity (DPPH method) after 7, 14, and 21 days (−40.16%, −49.61%, and −52.83%, respectively). Regarding the color parameters, b* and L* increased during 21-days storage, but no changes (*p* < 0.05) were reported for the color difference (ΔE). Microcapsules-added yogurts also exhibited a decrease in viscosity values, but this was attributed to capsule disintegration during storage and hydrogen bond formation between protonated carboxyl groups from alginate due to low pH.

Other dairy products have been formulated with natural colorants. Montibeller [117] assessed an ANC-rich extract from grape skin (Cabernet Sauvignon) on kefir, obtaining a decreased pH (up to ~4.55), increased acidity (up to ~0.080% citric acid), and low total soluble solids (~7.6 °Brix) after the addition of the extract in a 16-days evaluation of kefir performance. At the same time, L* and b* values increased (reaching 96.6 and 5.1, respectively), and a* progressively decreased to ~1.2. The storage time affected ANC retentions, but high values were obtained for peonidin-3-glucoside and delphinidin-3-*O*-*p*-coumarylglucoside (~77–88%). The authors suggested that the ANC-formulated kefir showed similar physical properties compared natural kefir without additives.

Ice creams are popular dairy or dairy-like frozen desserts at neutral pH, sometimes formulated with milk fat, milk proteins, fruits, and flavors. Stabilization of color in ice creams is still a problem that merits further research since some of them must be stabilized with polysorbate, negatively affecting the formation of overrun, one of the most desired attributes of ice creams [118]. The most common colorants used in ice cream are curcumin (intense lemon shade), Carthamus (bright yellow with slight greenish shade), β-carotenes (orange-yellow hues, preferred for vanilla-flavored products), annatto (yellow shades), beetroot (pink-red color), lycopene (red color), and chlorophylls or copper-chlorophylls (yellowish-green and bright green colors, respectively) [119].

Singo and Beswa [120] reported the impact of aqueous Roselle (*Hibiscus sabdariffa*) extracts (5%, 10%, 15%, and 20% *v/v*) on selected quality characteristics of ice cream. The extracts exhibited a direct relationship between the dose increase and L*, b*, and whiteness index, while a* progressively augmented up to 1.50 for the highest Roselle concentration. All Roselle-added ice creams showed lower L*, a*, and whiteness index values. Compared to a commercial vanilla-flavored ice cream control, overrun and melting rate values were higher (*p* < 0.05) (25.71–139.28% and 85.71–228.57%, respectively), but viscosity and pH were lower for the highest Roselle concentrations (*p* < 0.05, −2.97% to −3.13%; −4% to −6.22, respectively). The lowest Roselle-formulated ice creams (5% and 10%) showed no differences against the control in the sweetness and gummy taste evaluations. The authors considered that Roselle formula above 5% *v*/*v* would produce less viscous, high melting rate, unpopular color, and undesirable characteristics.

Durmaz et al. [121] used spray-dried microalga (*Nannochloropsis oculata*, *Porphyridium cruentum*, and *Diacronema vilkianum*) as coloring agents for ice cream (0.1, 0.2, and 0.3 g/100 g ice cream). The main pigments found in the spray-dried products were carotenoids (0.40–0.77 mg/g dry weight), chlorophyll *a* (1.06–4.76 mg/g dry weight), and ANC (2.34–23.96 mg C3G/kg). Compared to control ice cream, apparent viscosity decreased once added the microalga extracts, showing a resemblance to Newtonian fluids, being *P. cruentum*-added ice creams those with the most advantageous behavior as this microalga contains several carbohydrates such as cell storage polymers (starch derivatives), lipopolysaccharides, and extracellular polysaccharides. Ice creams enriched with *P. cruentum* displayed pinkish color, and the two other alga species showed a greenish color. Luminosity was not affected by type and concentration, while b* values increased together with alga concentration. Alga addition negatively impacted melting behavior, but the authors suggested the potential of optimization studies improving the ice cream composition and using bulking agents to overcome this situation.

Aqueous and ethanolic/methanolic betacyanin extracts from red pitahaya (*Hylocereus polyrhizus*) were applied as a colorant in ice creams [122]. The highest betacyanin yields (up to 18%) were acquired by adding pectinase (1.5% and 2.0% *v/v*) in the 95% ethanol extractions. During 21-day storage, betacyanins concentrations increased (~0.02%), and no significant color changes were found (*p* > 0.05). Betacyanin-supplemented ice creams exhibited the highest free radical scavenging activities (DPPH: 50–57%).

Using colorants in cheese is also a common practice. Natural colorants are preferred mainly for their health benefits, providing additional properties such as antioxidant, antimicrobial, and surface-active activity to colored cheese products [4]. The main colorant used in cheese and butter is annatto, but for the cheese industry, the colorant is mainly composed of norbixin, responsible for imparting yellow/orange color to cheddar cheese [123]. Other usual colorants used in cheese are carminic acid and ANC, paprika oleoresin, vegetable carbon, chlorophylls and chlorophyllin, and curcumin [80]. Saffron (*Crocus sativus* L.) was used as a colorant for fresh ovine cheese starting from a concentrated extract (1000 mg/L) and added to 2 L pasteurized ovine milk [124]. No differences were found for all treatments regarding moisture, total protein, salt, and fat contents (*p* > 0.05), saffron-added cheeses exhibited the lowest pH levels (4.13–4.36) and highest antioxidant capacity values (23.84–25.97% RSA). Saffron did not affect L*, but a* and b* values were higher compared to control cheeses. The 50 mg/L saffron-supplemented cheese was evaluated equally to control cheeses, while the highest saffron concentration negatively affected the sensory scores.

Sea buckthorn (*Hippophae rhamnoides* L.) fruit extracts were also assayed as colorants for cream cheese [125]. Chlorophylls (2.79 mg/L chlorophyll *a*; 4.73 mg/L chlorophyll *b*)), carotenoids (8.27 mg/L total carotenoids), and TPC (1842.86 mg/100 g dry weight) were the major quantified pigments and polyphenols from the fruits’ extracts. The addition of the extracts increased (2.04%) the average organoleptic score, decreased dynamic viscosity (up to 11258 mPa·s), and showed the same total viable count (4 × 10^2^ cfu/g) compared to 0.01% tartrazine-added cheese.

Lastly, dairy products are ideal complex food systems that can be used to test natural food-colorants properties since phenolics, and other components form interactions potentially reducing their abundance and health benefits. Hence, yogurt is one of the most tested dairy products to particularly test coloring properties and antioxidant capacity of their bioactive compounds [126]. Carotenoid and ANC are the most common colorant types used in dairy products, but the blue pigment provided by phycocyanobilins and the pH-stable shades given by betacyanins have opened an opportunity to these chemical groups to be more widely incorporated.

### 4.5. Meat and Meat Products

Curing is a highly valued process in the meat industry since it prevents *Clostridium botulinum* growth and development. Concerns about this process’ carcinogenic and toxic effects of nitrosamines as a result of the nitrite and nitrates have stimulated research in other colorants not only for a generation of a stable color but to reduce the need of using curing salts. In this sense, it has been found specific applications for plant-derived colorants such as beetroot (*Beta vulgaris* L.: red betacyanins and yellow betaxanthins), paprika (*Capsicum annuum* L.: red color), tomato (*Solanum lycopersicum*: lycopene); and microbial pigments like pigments from *Monascus purpureus* (purple color) [127].

Slightly colored meat products (e.g., pork and turkey) are some of the most routinely used food systems to evaluate the pigment properties of natural colorants. Several researchers have focused on the assessment of these pigments in sausages, widely consumed worldwide. Microencapsulated jabuticaba (*Myrciaria cauliflora*) extract (2% and 4% *w/w*) was added to fresh sausages [128] and the resulting product showed the same (*p* > 0.05) proximal composition, lower (*p* < 0.05) thiobarbituric acid reactive substances (TBARS) development (0.01–0.05 mg malonaldehyde, MDA/kg sample, compared to 0.39–0.60 mg MDA/kg), and major color preservation compared to control and carmine-formulated sausages. Purified fucoxanthin from Tunisian seaweed (*Cystoseira barbata*) allowed reductions from 150 to 80 ppm in the nitrite concentration of turkey-meat sausages due to enhanced color preservation and improved oxidative stability, but no antimicrobial evaluations were carried out, one of the main purposes of using nitrites [129]. Similar sausages were formulated with carotenoproteins from blue crab (*Porturus segnis*) shells (84.44% yield, 1211–1135 μg GAE/g extract) [130], where the developed sausages exhibited a 10-day shelf life with decreasing diene formation, metmyoglobin, and heme iron preservation, and improved DPPH radical scavenging than control sausages.

The evaluation of colorants can also be used in cooked meat to preserve color and avoid lipid oxidation. Both complex processes still a major concern in loss of sensory quality, nutritional properties, and economic value [131]. Astaxanthins from *Haematococcus pluvialis* (20 mg/kg, 40 mg/kg, 60 mg/kg, and 80 mg/kg) showed strong antioxidant properties when applied to fresh, frozen, and cooked lamb patties [75], showing pH preservation, lower TBARS levels than control patties (no antioxidants; and low L*, higher a*, and higher b* values than control patties. More recently, Cunha et al. [132] reported the antioxidative properties of encapsulated pitaya (*Hylocereus costaricensis*) peel extract (100 and 1000 ppm) on pork patties subjected to high-pressure processing, manifested in slight L* and b* increases, a* preservation, color differences closer to 1, preserved cohesiveness and springiness, and low concentrations of MDA along time.

As meat products are mainly associated with reddish and orange colors, carotenoids have especially found a niche in these food products. Moreover, since nitrites and nitrates are applied into these products to take advantage of the antibacterial properties, using nitrogen-based natural food-colorants could be an alternative to reduce the amount their amount, preventing health concerns associated to their use. Thus, betacyanins could be one of the most potential colorants to be used in these food systems, but process extraction and optimization is needed to test their curing ability, nitrosamine formation, and their ability to produce a desirable color.

### 4.6. Other Food Products

Natural colorants are widely used in pasta products to produce new ways of colored pasta, especially the popular “vegetable-added pasta”. Dalla Costa et al. [133] used 20% carrot (*Daucus carota* sbsp. sativus) flour as a substitute for β-carotene for commercial dry wheat (*Triticum aestivum*) pasta and found 307% higher levels of carotenoids, 132% increased antioxidant capacity, and 608% higher total fiber compared to no carrot-added pasta. Saffron (*Crocus sativus* L.) enrichment (0.2–0.4% *w/w*) of wheat flour pasta [134] decreased L* but increased a* and b* values compared to commercial pasta, and no textural parameters were affected (hardness, cohesiveness, elasticity, and chewiness). In contrast, saffron allowed higher antioxidant capacity values (DPPH: 4–6 μmol Trolox equivalents/g dry base vs. 0.5–4 μmol Trolox equivalents/g dry base in control pasta). Panelists positively scored saffron-added pasta in terms of aspect, color, aroma, taste, and global acceptability.

The addition of “Senduduk” fruit (*Melastoma malabatrhicum* L.) (2–10%) to jackfruit jam enhanced β-carotene (300–314 g/100 mL), ANC (6.86–9.43 mg/L), TPC (0.99–1.34 mg/mL), and antioxidant capacity [135].

Cerezal Mezquita et al. [136] assayed lutein obtained from *Muriellopsis* sp. alga biomass as a natural and antioxidant in a mayonnaise-like dressing sauce. Prepared mayonnaises showed high pigment stability in the matrix based on the L*, a*, and b* values. The tested mayonnaise showed similar moisture and lipids than corn oil mayonnaise and higher lutein than commercially available mayonnaises.

Finally, the plenty applications of natural food-colorants demonstrate their potential to be incorporated in several food systems beyond the traditional formulations. New sources are constantly being incorporated as sources of colorants after an optimization procedure to overcome not only technological aspects, but also legal and toxicological concerns, and the consumers’ attitudes towards these colorants. A summary of all technological applications of natural colorants in food systems is shown in Table 2. 

## 5. Conclusions and Perspectives

The use of natural colorants in food systems is still limited due to technological issues. Alternative sources of colorants should be explored, aiming to find more stable, physicochemical feasible, and improved color stability from traditional and novel sources. Underutilized tropical fruits and vegetables such as Andean, Amazonian, and South-Asian products are still underdeveloped raw materials to extract valuable natural food-colorants. Emerging technologies such as ohmic heating and EF-based technologies have the advantage of using less energy and water for extracting compounds, whereas PEF easily induces electroporation on the food matrix, accelerating the extraction to reach <80% yield for some colorants. For heat-labile compounds, HPE allows the extraction without using temperature, preserving its functional characteristics. However, extraction technologies should be optimized to provide environmentally feasible and low-cost colorants from the actual and novel sources.

Although the addition of some of these novel ingredients might affect the physicochemical properties of these products, as shown in dairy and dairy-like products such as ice creams, optimization procedures improving the existing formulations could positively enhance the inclusion of these nutritionally rich ingredients. Hence, new textures and sensory outcomes could be provided, together with a nutritional advantage derived from the health-associate properties of most of these colorants. Understanding the chemical composition of the natural food-colorants and their interaction with the food matrix is a key factor to manufacture food products with the desired color stability. More research is needed to stabilize most of these colorants at the varied range of the usual pH and temperature conditions in the intended food systems.

## Figures and Tables

**Table 1:** Main outcomes from reported industrial extraction technologies of natural colorants.

Extraction Technology	Main Outcomes	Reference
Ohmic heating (OH)	Aqueous extraction of phenolic compounds extracted from wheat bran. The best conditions were 20 V/min, 80 °C, 10 min holding time to obtain 3150 mg/kg of phenolics and 82% antioxidant capacity.	[44]
	Aqueous extraction of ANC with a yield > 80% from blue potato. Maximum recovery at 15 V/90 °C/10 min.	[40]
	Dark purple ANC were extracted from black rice bran with a higher yield (20.63%) using OH compared with steam extraction. Conditions used at 30% and 40% moisture and 100–200 V/cm (105 °C, 1 min).	[45]
	Polyphenols extraction was accelerated with OH due to higher cell wall disruption. Higher yield (36%) was achieved with 400 V/cm with 30% ethanol-water.	[41]
	Ethanol-water polyphenolic extracts were obtained from vine pruning residue. At 840 V/cm, 80 °C and 60 min extraction, antioxidant, antimicrobial and anticancer activity were observed.	[48]
	ANC have a high rate of degradation after OH application in blueberry pulp.	[46]
	OH treatment was used on fungal red colorant in a beverage model system. Pigment degradation of 33% was observed with OH compared with 23% with a conventional method.	[49]
OH, and microwave-assisted extraction	Development of several hybrid drying methods used to obtain red beetroot powder	[72]
Pulsed electric fields (PEF)	A response surface model was used to obtain the optimal values for ANC extraction using PEF. Optimal extraction (166 mg ANC) was found at 15.08 kV and four pulses.	[52]
	PEF was applied as pretreatment induced cell permeabilization and higher ANC yield. Maximum recovery (65.8 mg/100 g ANC) was achieved at 3.4 kV/cm, 105 ms pulses, 40 °C, and 480 s processing time.	[53]
	PEF treatment allow a “cold” extraction at low temperature (30 °C) with 95% yield and 10% colorant degradation. The conditions used were: 0.375–1.500 kV/cm; 120 pulses (100 ms), 30–80 °C.	[54]
	PEF-assisted extraction of astaxanthin from *Haematococcus pluvialis* was performed at 0.2–1 kV/cm, 10–80 pulses of 5 ms for 6 h. Methanol and ethanol improved the extraction. A further aqueous incubation was necessary to recover the colorant.	[55]
	Cell permeabilization caused by PEF pretreatment, allows nearly 100% b-phycoerythrin extraction from the alga *Porphyridium cruentum*. For this experiment, 10–50 pulses of 3 μs at electric field 2–10 kV/cm, room temperature were used.	[56]
	PEF was used to increase the stability of chlorophyll previously extracted with ethanol from *Spinacia oleracea* L. The maximum recovery was observed at 26.7 kV/cm, 35 °C and 0.32 ms.	[57]

ANC: Anthocyanins.

**Table 2:** Technological applications of natural colorants in food systems.

Product	Pigment Origin	Obtention Method and Experimental Procedure	Technological Applications	Ref.
**Bakery Products**				
Cupcakes	Roselle (*Hibiscus sabdariffa* L.)	ANC-rich extract (delphinidin-3-sambubioside, cyanidin-3-sambubioside, and delphinidin-3-glucoside). The extract was obtained by drying Roselle calyces (28 °C, 3 h), followed by ground (0.55 mm) and soaking in water (200 mL). The suspension was heated at 80 °C for 1 h.	Improved proximal composition (higher dietary fiber and ash than control cupcakes), pinkish crumb and crust color, preservation of several sensory parameters (color, appearance, texture, taste, volume, and aroma).	[73]
French macarons	Jabuticaba (*Myrciaria jaboticaba* (Vell) Berg)	ANC-rich jaboticaba epicarp extract was obtained after optimized heat- and ultrasound-assisted extraction (21.8 min, 47.1 °C, 9.1% ethanol *v/v*; 7.49 min, 421.82 Watts, 48.30% ethanol *v/v*, respectively).	Proximal composition and color stability up to 6 days was obtained. Formulated cupcakes presented high TAC (81 ± 2 mg/g), being C3G and D3G the most notorious ANC.	[81]
Cake and cookies	Tomato waste	Lycopene was extracted from tomato waste using several temperatures (20, 30, and 40 °C) and extraction times (15, 30, 45, and 60 min) using 25:75 acetone:n-hexane ratio. Once the solvent was removed by evaporation (50 °C), the resulting lycopene was used (81.75–93.59% recovery yield).	Improved antioxidant capacity (measured by DPPH). Lycopene-added cakes and cookies showed higher volume and increased L*, a*, and b*, but there was no impact on the overall acceptability.	[26]
Water biscuits	Red beetroot (*Beta vulgaris* L., cv. “Bicor”)	Beetroot pomace was separated by vacuum filtration of the juice. Solvent extraction was then conducted for the pomace (83.3:16.7 ethanol:0.5% acetic acid proportion). After ultrasound treatment (30 min, 24–25 °C, water bath), centrifugation (9000 rm, 10 min), the solution was vacuum-filtered and vacuum-concentrated (35 °C), yielding 6.87 g dry matter/g.	Red beetroot-added biscuits showed increased betanin and isobetanin contents (up to ~55 mg/kg DM), TPC (up to ~2300 mg GAE/kg DM), and antioxidant capacity (FRAP and ABTS) compared to untreated biscuits.	[9]
Wafers	*Arbutus unedo* fruit	A C3G-rich *A. unedo* extract was prepared using heat-assisted extraction. Briefly, 600 mg sample was mixed with acidified ethanol (80% ethanol acidified with 0.05% HCl), stirred (500 rpm, 5 min, 90 °C), and filtered (Whatman n°. 4 paper). A residual extract yielding 60% of the total fruit dry weight and 500 μg/mL ANC was obtained.	Extract added wafers only showed a significant a* changes (*p* < 0.05) after 3- and 6-day storage. Compared to control wafers, higher sucrose, fatty acids contents, and antioxidant capacity.	[79]
Donuts	Blackberry (*Rubus ulmifolius* Schott)	Optimized blackberry ANC-rich extract was obtained using heat-assisted extraction and a RSM analysis. One gram of the fruit was mixed with 20 mL ethanol acidified with citric acid. The solid to liquid ratio was maintained at 50 g/L. The samples were then centrifuged (6000 rpm, 20 min, 10 °C), and filtered (Whatman paper filter n° 4).	Compared to control donuts, L* and b* were lower, but a* was higher. Free sugars (fructose, glucose, sucrose, and trehalose) decreased along storage time (3 days), and no differences in free fatty acids were obtained.	[76]
**Beverages**				
Alcoholic beverages (up to 30% alcohol)	*Porphyridium* sp. microalga	Fluorescent phycobiliproteins (240 kDa molecular weight, λ: 545–575 nm). Obtention after water or buffered solution extraction, centrifugation, microfiltration, and freeze-drying.	Yellow color, stable at pH 5.0–6.0	[1]
No-heat treated carbonated beverages	*Porphyridium aerugineum* microalga	C-phycocyanin (λ: 620–642 nm). Color obtained after centrifugal separation of algae biomass, salt extraction, microfiltration, or co-precipitation of polysaccharides.	Color stability at pH 4.0–5.0 for at least 1 month at 25 °C, 40 min at 60 °C. The pigment was successfully assayed in Pepsi^®®^ Blue.	[1]
Green tea model beverage	Purple carrot	ANC solution (0.05%) with 20 mM calcium hydroxide until reaching 0.02%, prepared at pH: 3.0	Improvement of color stability from ANC (2.62–6.73 days), even better at higher temperatures (25–40 °C).	[13]
Sports beverage	Black bean (*Phaseolus vulgaris* L.) seed coat	Seed coats were subjected to an aqueous extraction (40 °C, 4 h), pH-adjusted with citric acid (2.0), centrifuged (27,200× *g*, 15 min), filtered, and stored at -20 °C (ANC-rich extracts). For their addition to a commercial sports beverage, extracts (0.1 mg/mL or 0.26 mg/mL) were added to 250 mL of a commercial glacier cherry-flavored sports drink. β-Cyclodextrin was then added to reach 2 g/100 mL concentration.	ANC extract-added beverages co-pigmented with β-cyclodextrin exhibited longer half-life, similar lightness, lower a*, and higher b* than commercial sports beverages.	[87]
Model commercial beverages	Pitaya (*Stenocereus pruinosus*).	Pitaya was collected, homogenized (1 g), mixed with 4 mL water, vortexed (3150 rpm, 1 min), and centrifuged (10,576× *g*, 20 min), and supernatants were recovered.	Yellow beverages displayed several yellow-orange shades. Juice-addition (5%) showed similarity with commercial beverages, retaining up to 75% of total betaxanthins.	[34]
	Yellow bell pepper (*Capsicum annuum* L.)	Ripe yellow bell peppers were dried (55 °C, 15 h), powdered, and pigments were extracted after alcohol maceration with ethyl alcohol and water (90:10 *v/v*). Hexane partition was carried out, and the organic solvent was evaporated (40 °C, vacuum rotary evaporator). Inclusion complexes with β-cyclodextrin were prepared (1:2, 1:4, and 1:6 *w/w*) using ultrasound-freeze drying and molecular inclusion.	L* and a* parameters increased together with extract concentration, but b* decreased in the tested beverage models.	[88]
	Yellow-orange cactus (*Opuntia ficus-indica*)	Cactus pulp was vacuum-concentrated (30 °C, 17 mbar) up to 45 ºBrix. For the freeze-dried extract, maltodextrin was added (1:1 pulp:maltodextrin), homogenized, frozen (−50 °C, 48 h), and dried (−55 °C, 0–0.133 mbar).	Betaxanthin-rich extracts contained 256.53–264.76 mg indicaxanthin equivalents/kg. Soft-drink beverages displayed significant color changes after a 5 days-storage (4 °C).	[89]
	Annato from *Bixa orellana* L. seeds, gardenia yellow from *Gardenia jasminoides* Ellis, lutein from marigold flowers or *Tagetes erecta* L., curcumin from turmeric or *Curcuma longa* L., and safflower extract from *Carthamus tinctorius* L.	All colorants were acquired locally from commercial manufacturers. Beverages were formulated with McIlvaine buffer (pH: 3.5, 5.5, and 7.5; concentration: 0.001%, 0.005%, 0.01%, 0.02%, 0.03%, 0.05%, 0.10%, and 0.30%) with and without ethanol (15% *v/v*).	Gardenia, safflower, and curcumin displayed the highest color intensities and lowest turbidity levels. Safflower colorant was the most heat- (25–80 °C) and light-stable (550 Watts/m^2^, 30 °C).	[91]
	Purple corn (*Zea mays* L.) pericarp	ANC and flavones were extracted in a 1:2 ratio (*w/v*) from the corn seeds after aqueous incubation (80 °C, 1 h) under constant shaking. After cooling, extracts were filtered (Whatman n° 1 paper) and stored frozen at −80 °C.	Flavone addition increased the average half-life of cyanidin or pelargonidin-rich model beverages, but cyanidin beverages were the most stable ones.	[92]
Protein beverage	Jabuticaba (*Plinia cauliflora*)	Jabuticaba skins (40 g) were ground, mixed with 70% *v*/*v* acidified ethanol with citric acid (pH: 2.0), and left to stand for 24 h (5 °C). The extract was vacuum-filtered (Whatman n° 1) and concentrated in a vacuum rotary evaporator (40 °C). The extract was added to whey (0.5%, 2.0%, 4.0%, and 6.0%)-based beverages, formulated with mineral water, sugar (15% *w/v*), strawberry pulp (10% *w/v*), gum arabic (0.45% *w/v*), potassium sorbate (0.03% *w/v*), and citric acid.	D3G and C3G were the main ANC from the extract. Formulated beverages showed whey concentration-dependent TPC (32.6–83.6 mg GAE/100 g) and antioxidant capacity (1.2–1.8 μM TEAC/g) values	[93]
Jelly drink	Purple sweet potato (*Ipomoea batatas* L.) cv. Ayamurasaki	ANC were extracted from purple sweet potato and encapsulated with 6% *w*/*v* maltodextrin. The jelly drinks contained 0.3% *v*/*v* jelly powder and 12% *v*/*v* sucrose dissolved at 75 °C for 5 min. Potassium citrate and sodium benzoate were added, and the product was cooled at 40 °C.	Beverages stored at 5 °C without light exposure presented the lowest ANC and b* decrease, and average shelf-life of 200 days.	[94]
**Confectionery**				
Gummies	Pitaya (*Stenocereus pruinosus*).	Pitaya was collected, homogenized (1 g), mixed with 4 mL water, vortexed (3150 rpm, 1 min), and centrifuged (10,576× *g*, 20 min), and supernatants were recovered.	Betaxanthins were reduced by half after 11 days of storage at 40 °C. Gummies showed high variations in yellow to orange color.	[34]
	Cactus fruit (*Opuntia ficus-indica*) (purple pulp)	Betalains-rich extracts were obtained by crushing cactus fruit pulp and removing seeds by filtration. The product was then freeze-dried (1.9–2.3 g/100 g final moisture), and macerated with phosphate buffer (pH 5.5, 1:2 pulp:buffer ratio). The betalain-rich extract was mixed with sodium alginate (15 g/L, pH: 5.5), slowly added to calcium chloride solution (0.015 M) for 1 min, and washed with distilled water. The obtained beads were then dehydrated (30 °C, 24 h, forced-air circulating oven).	Gummies showed no significant *(p* > 0.05) variations in color during 30 days of storage at 4 °C. Vivid red-purple color gummies were obtained.	[99]
Condensed milk-based confections and doughnut icing	Fig (*Ficus carica*) and blackthorn (*Prunus spinosa* L.)	Peel from *F. carica* and epicarp from *P. spinosa* were freeze-dried and milled (20 mesh size). Ultrasound-assisted extraction was conducted: 100 mL acidified ethanol (figs: 180 g/L, 21 min, 310 W) or 50:50 ethanol:water (blackthorns: 75 g/L, 5 min, 400 W). Samples were then centrifuged (6000 rpm, 20 min, 10 °C), filtered (Whatman n° 4), and supernatants were freeze-dried. Cyanidin-3-rutinoside-rich fig and cyanidin-3-rutinoside/peonidin-3-rutinoside-rich blackthorn extracts were obtained.	Formulated products mainly contained sucrose, palmitic acid, and mostly saturated fatty acids due to dairy ingredients. Blackthorn-added samples were the darkest one (purple color).	[98]
Gummy model	Saffron (*Cocus sativus*) and beetroot (*Beta vulgaris*)	Saffron (1 g) was extracted with water under constant shaking in a water bath (25 °C, 60 min, 30 kHz). Beetroots were washed, peeled, and extracted with water using a commercial juice extractor. Both extracts were microencapsulated using blends of gum arabic, modified starch, and chitosan, and mixtures were encapsulated by freeze-drying (0.017 mbar, −57 °C, and 48 h).	Storage temperature (25 °C and 40 °C) decreased luminosity, a*, and b* values for both extracts. The gum arabic and modified starch mixture exhibited the highest color stability: a* (for beetroot-added gums) and b* (for saffron-added gums).	[100]
Chewy candy	Açaí (*Euterpe oleracea* Mart.) pulp	Frozen Açaí pulp was thawed (25 °C), maltodextrin was added (60 g/100 g), and the mixture was homogenized (200 L/h, 10 HP). The powder was obtained by spray-drying (0.5 mm diameter nozzle and 6000 rpm atomizer, IAT: 170 °C, OAT: 80 °C, flow rate: 10–15 kg/h). This powder was added to candies prepared in an atmospheric batch system cooker.	The Açaí-added candies did not exhibit differences in the hardness or moisture content, presented higher color acceptance, and high purchase potential (from uncertain panelists), compared to non-added Açaí candies.	[102]
Hard-panning confections	Uvaia (*Eugenia pyriformis*)	The Uvaia by-product (peels and seeds) was thawed, centrifuged, and oven-dried (40 °C, 24 h). Seeds were removed, and peels were milled (particle size: 37 μm). The powder was added to hard-panning confections made after cooking gummy candies (110 °C), adding starch, and following sealing and panning stages.	Uvaia-added candies showed the highest a*, b*, hardness, the best appearance, and color sensory scores, but the lowest crispness, compared to fruit concentrate-added and artificial colorant-added candies.	[105]
Jelly gummy candies	Black Elderberry (*Sambucus nigra*)	*S. nigra* dyes obtained from fruits, flowers, and their mixture were freeze-dried (100 g of raw material mixed with 200 mL water, boiled for 10 min, frozen at −60 °C, and lyophilized). The obtained powder was dehydrated (48 h in heating shelves at 30 °C, 0.5 bar pressure). Jellies made from gelatin, Agar, and honey were used to add the powdered dyes.	Extract-added jelly gummy candies contained ANC such as cyanidin.3-*O*-sambubioside-5-glucoside, cyanidin-3,5-diglucoside, cyanidin-3-*O*-sambubioside, and cyanidin-3-*O*-rutinoside, and high antioxidant levels measured by FRAP and DPPH.	[106]
White chocolate	*Nannochloropsis oculata* microalga	Method 1: Algal biomass was dried in a spray-dryer (6 bar, 1.40 mL/min flow, and 65 mbar atomization pressure). IAT: 180 °C, OAT: 95 °C. 1:1. Method 2: Alga:maltodextrin proportion was mixed (10,000 rpm, 10 min). Encapsulation was carried out in a freeze-dryer. For adding the encapsulated products to white chocolate (6 h, 60 °C conching time), alga powders (0.125, 0.25, 0.50, and 0.75 g/100 g) were added on the last 15 min of the conching process.	The resulting alga-added chocolate exhibited higher a* and hue values than the control white chocolate samples. Chlorophyll *a* values ranged from 9.60 to 27.2 μg/g. No significant differences (*p* < 0.05) were shown for the sensory analysis of appearance, texture, and smell, despite being evaluated with lower values than the control chocolate samples.	[108]
Transparent lollipops made from sugar solutions Dry sugar-drop candies for cake decoration	*Porphyridium* sp. microalga	Fluorescent phycobiliproteins (240 kDa molecular weight, λ: 545–575 nm). Obtention after water or buffered solution extraction, centrifugation, microfiltration, and freeze-drying.	Pinkish-red color on confections, stable at 60 °C for 30 min, and long shelf-life (6 months) at pH 6.0–7.0	[1]
**Milk, dairy, and dairy-like products**				
Yogurt	Ayrampo (*Opuntia soehrensii* Britton and Rose) seed	Betalains were obtained by soaking the seeds in distilled water (pH: 4.5, acidified with 0.25 N HCl, 1:3 *w/v*) for 24 h at 30 °C. Samples were then centrifuged (4000 rpm for 15 min), and supernatants were collected and filtered (Whatman paper n° 4). The purification was carried out by gel filtration chromatography (Bio-Gel P-2 columns) using a freeze-dried liquid-liquid extract with ethyl acetate (4:1 solvent:betalain extract) at pH: 4.5 (12 h). The fractions were eluted with distilled water (6.8 mL/h).Betacyanins were extracted by mixing the seeds’ extract with McIlvaine buffer (0.15 M, pH: 5.6) until obtention of absorbance between 0.2 and 0.8 (537 nm).	Betacyanin-added yogurts showed lower L* and higher ΔE than control yogurts, but the 5-week storage showed similar performance than the synthetic colorant Red no. 40 in color retention (>94%) and L* values.	[111]
	Curcumin (*Curcuma longa*)	Commercially acquired curcumin (10 mg) was mixed with Tween 80 (10 mg) and stirred for 5 min. After sonication (15 min) under pulse conditions (30 s, 120 W, 25 °C), the solvent was evaporated (40 °C, 24 h), and the solid was ground with pistil and mortar (8.30% *w*/*w* curcumin was obtained). Different proportions of natural curcumin and encapsulated curcumin were added to commercial natural yogurts.	Formulated yogurts showed color ranges closer to orange (mango, peach, or papaya-like color). During 7-day storage, a* and b* values decreased compared to control yogurts, but the overall color was maintained a long time.	[112]
	Petals of *Dahlia mignon*, rose from *Rosa damascena* “Alexandria” and *Rosa gallica* “Francesca”; and flowers from *Centaurea cyanus* L.	Flowers were reduced to powder (20 mesh), and 1 g of the dry material was mixed with 50 mL of distilled water to be extracted by maceration (25 °C, 150 rpm, 1 h). Mixtures were filtered with Whatman Paper n 4, frozen, and freeze-dried. Commercial yogurts (3.8% fat) were supplemented with *Dahlia* (0.05% *v/v*), rose (0.15% *v/v*), or *Centaurea* (0.10% *v/v*) extracts.	Manufactured yogurts exhibited the same proximal composition and color parameters as artificially-colored yogurts (E163) but showed a higher monounsaturated fatty acids composition (oleic acid).	[113]
	Jabuticaba (*Myrciaria jaboticaba* (Vell) O. Berg) and *jamelão* (*Syzygium cumini* (L.) Skeels)	Fruits were washed, and peels were manually separated from the pulp, dried (60 °C, air speed: 1 m/s, 22 h). The dried product was ground and used to formulate yogurts (0.3 and 0.5% *v/v*).	Jabuticaba-colored yogurts displayed better appearance, flavor, and color scores than *jamelão*-colored yogurts (*p* < 0.05). No differences were found for the appearance between not-colored and jabuticaba-formulated yogurts.	[78]
	Strawberry (*Fragaria ananassa*)	ANC from Strawberries were extracted after mixing strawberry (0.5–2.0 g) with 85% distilled water and 15% HCl (0.1 M) (pH: 1.3) under agitation (400–800 rpm, 1–15 min), followed by centrifugation (2486× *g*), and filtration (13 μm).	ANC-addition produced yogurts with 10–40 mg/100 g TAC and a remaining red color at pH: 4.6 (yogurts’ pH) and 4 °C storage.	[114]
	Red beetroot (*Beta vulgaris* L.), opuntia (*Opuntia stricta*), Roselle (*Hibiscus sabdariffa*), and radish (*Raphanus sativus* L.)	Betalains-rich extracts (red beetroot and opuntia) were prepared using small hand-peeled raw materials pieces (5 g) and adding a water:ethanol:acetic acid (66.6:33:0.33 *v/v/v*) solution (25 °C) for 48 h (beetroot) or 20 min (opuntia). Mixtures were filtered and centrifugated (500 rpm, 16 min), and solvents were evaporated by rotary evaporation (40 °C). The ANC-rich extract (Roselle) 5 g of flowers were mixed with a water:ethanol:acetic acid (70:29.7:0.3 *v/v/v*) solution (4 °C, 72 h). The mixture was filtered, and the solvent was evaporated (40 °C, rotary evaporator). ANC-rich extract from red radish was obtained by making blends of radish (25 g) with water/acetic acid (95:5 *v/v*) (100 mL), and the solution was kept at 4 °C for 18 h. After filtration, the solvent was evaporated. All extracts were freeze-dried (−80 °C, 5 days), nanoencapsulated in liposomes, and applied to soy-based yogurt alternative.	Yogurts contained betacyanins, ANC, or betalains accordingly to the origin of their extracts. High color retention was observed after 21 days of storage, but Roselle and red radish-origin colorants were the most stable.	[115]
Fermented flavored milk	Canthaxanthin from *Dietzia natronolimnaea* HS-1	*D. natronolimnaea* HS-1 was transferred to a 100 mL liquid-pre-culture medium (10 g/L glucose, 5 g/L peptone, 5 g/L yeast extract, and 3 g/L malt extract). Then, the inoculum was transferred to another medium (10 g/L yeast and 40 g/L beetroot molasses) and incubated (28 °C, 180 rpm for 5 days). The biomass was removed by centrifugation (8000× *g*, 5 min), washed with physiological serum (9% NaCl), and extracted with ethanol by centrifugation (8000× *g*, 10 min). The pigment was microencapsulated using oil/water/oil multiple emulsion external gelation. Capsules were applied to pasteurized or flavored fermented milk samples (15 mg/L).	Colorant-added yogurts retained less than 50% of antioxidant capacity after 21-day storage. No differences in ΔE were shown between the formulations and a reference yogurt.	[116]
Kefir	Grape (Cabernet Sauvignon)	Grapes’ husks were manually separated and stored at -18 °C. Then, 25 mL of acetate buffer (pH: 4.0) was added to 5 g of frozen husks, heated at 40 °C, and stirred (150 rpm, 30 min). The resulting extracts were freeze-dried (−55 to 57 °C, 200 μHg, 4 days) to obtain ANC-concentrated extracts. Extracts were added to the prepared fermented product from kefir (400 mL ANC extract + 2 L kefir).	pH, L*, and a* decreased during 16-day storage, compared to initial values. High ANC retentions were obtained at the same time (77–88%). ANC-added kefir exhibited similar physical properties as natural kefir.	[117]
Ice cream	Roselle (*Hybiscus sabdariffa*)	Fresh Roselle calyces were washed and dried (50 °C, 36 h) in a hot-air oven dryer, powdered (0.8 mm particle size), and mixed with proper amounts of deionized water to achieve 5%, 10%, 15%, and 20% *v/v*. Mixtures were soaked in a water bath (75 °C, 1 h), filtered (Whatman paper n° 1), and residues were extracted with 300 mL water as described.	5% Roselle-added ice creams displayed the best viscosity (242.3 cP), melting rates (1.3 g/min), and color attributes (L*: 72) among the formulations. Moreover, the lowest Roselle-added (5% and 10%) ice creams displayed no differences (*p* < 0.05) in the sweetness and gummy taste compared to commercial vanilla-flavored ice cream.	[120]
	Microalga (*Nannochloropsis oculata*, *Porphyridium cruentum*, and *Diacronema vilkianum*)	Microalga was cultured in F/2 culture media prepared with seawater (350 g/L salinity, pH: 7.5, 25 °C, 2% CO_2_), and biomasses were harvested, concentrated, and dried in a spray-dryer (1.0 m nozzle diameter, AIT: 70 °C, OAT: 95 °C, 7–9 mL/min feed rate, residence chamber: 1.5 s). The spray-dried product was mixed with ice cream mix (0.1, 0.2, and 0.3 g/100 g ice cream) by centrifugation (1300 rpm, 3 min), followed by rapid colling at 4 °C. Samples were aged 24 h at 4 °C, whipped (0 °C, 10 min), and frozen at −18 °C for 24 h.	Formulated ice creams exhibited lower apparent viscosity and lower performance of melting behavior compared to control ice creams. *P. cruentum* provided a pinkish color, while the other two microalgae exhibited a greenish color. TPC were higher (*p* < 0.05) than the control ice creams, particularly for *N. oculata* alga (up to ~225 mg GAE/kg ice cream). No differences were shown between the *P. cruentum*-added ice creams and color, texture, taste, odor, resistance to melting, mouthfeel, or overall acceptability.	[121]
	Red pitahaya (*Hylocereus polyrhizus*)	Betacyanins were extracted from the pulp using distilled water, 50% ethanol, or 95% ethanol in a 1:1 or 1:2 fresh weight:solvent ratio (*w/v*). Pectinase (0, 0.5%, 1.0%, 1.5%, 2.0%, or 2.5%) was used to degrade the pectin. The pulp was then homogenized (2 h, 15,000× *g*, 15 min), and supernatants were placed on a vacuum oven for 24 h. Extracts (50 mg/mL) were added to fresh cow milk and pasteurized (63 °C, 30 min). After cooling (4 °C), a commercial powdered ice cream pre-mix (2% fat) was used, and the mixture was placed in an ice-cream maker. The resulting ice cream was frozen at −18 °C.	The betacyanin concentration and free radical scavenging activity increased during 21-day storage in the supplemented ice creams. No sensory evaluations were conducted.	[122]
Cheese	Saffron (*Crocus sativus* L.)	Saffron flowers (0.5 g) were ground and added to 0.5 L of milk (1000 mg/L) at 42 °C under slow agitation for 45 min. The mixture was filtered (500 μm mesh) and used in the cheese trials. For the cheese, ovine milk (8 L) was pasteurized (68 °C, 10 min), the milk was cooled (30 °C), and inoculated with a starter culture (10^8^ cfu/mL at 1% rate: 3.50 × 10^6^ cfu/mL). Saffron extract (100, 150, and 200 mL of the extract), commercial rennet, and salt were added. Mixtures were incubated (25–28 °C, 12 h), mixtures were set in cheese-cloths, ripened (25 °C, 6 h), drained, and stored (4 °C).	The saffron addition did not affect moisture, total protein, salt, and fats, but these cheese showed the lowest pH (4.13–4.36) and the highest antioxidant capacity values (up to 25.97% RSA). Cheese with the lowest saffron concentration (50 mg/L) received the same sensory score as control cheeses.	[124]
	Sea buckthorn (*Hippophae rhamnoides* L.) cv. “Elizaveta” fruits	Cylindrical fruits with a sweet-sour taste were powdered (particle size: 85 μm), mixed with deodorized refined sunflower oil (1 g extracted with 12 mL of oil), stirred, and sonicated at two different temperatures (20 °C and 45 °C) and three extraction times (0.5 h, 1.0 h, and 1.5 h). The extracts were centrifuged (7000 rpm, 10 min), decanted, and stored at 4 °C in dark glass bottles. The extracts (2.2% of cheese’s mass) were added to manufactured cream cheeses at 20 °C, homogenizing the samples for 5–10 min.	Manufactured cheeses incorporated chlorophylls, carotenoids, and TPC from the fruits’ extracts and received better sensory scores than tartrazine-supplemented cheeses.	[125]
**Meat and meat products**				
Sausages	Jabuticaba (*Myrciaria cauliflora*)	Residues from Jabuticaba fruit (peels and seeds) were mixed with water (1:3 residue:water) under mechanical agitation (6 h). The fluid was filtered and concentrated to 1/3 of its original volume (rotary evaporation: 60 °C under vacuum). The extract was mixed with maltodextrin, stirred, and microencapsulated in a spray dryer (atomizing nozzle diameter: 1.5 mm, IAT: 150 °C, 40 L/min airflow, and 30 mL/min feed rate). Extracts (2% and 4% *w/w*) were added to manufactured sausages (pork shoulder and backfat, NaCl, condiments, and Na_5_P_3_O_10_).	No differences in the moisture, protein, lipids, or fat (*p* > 0.05) were found between all formulations. Jabuticaba-formulated sausages exhibited low TBARS formation (0.01–0.05 mg MDA/kg sample), L* (57.5–63.4), a* (5.7–9.1), and b* (4.8–11) changes during 15-days storage, compared to control sausages. Only 2% *v*/*v* manufactured sausage showed the same overall acceptance as control and carmine-added sausages.	[128]
	Brown seaweed (*Cystoseira barbata*)	Brown seaweed was collected, water with seawater and tap water (25 °C), dried (20 days), milled (0.2 mm mesh size), and stored in amber glass bottles at 4 °C. Fucoxanthins were extracted by mixing the algal powder (100 g) with acetone:methanol (7:3 *v/v*, 24 h, 30 °C) under stirring (250 rpm). Extracts were concentrated and redissolved in 100 mL methanol, mixed with 300 mL water and 400 mL diethyl ether. The upper phase containing the pigment was collected, dried in a rotary evaporator, and dissolved in 5 mL of N-hexane. Silica gel column chromatography was used to purify the pigment.	Fucoxanthins-added sausages showed less L*, but higher a* and b* values than control sausages. The reddish color was improved compared to 150 ppm sodium nitrite and vitamin C references. Sausages containing fucoxanthin exhibited less TBARS formation compared to 80 ppm sodium nitrite formulated sausage.	[129]
	Blue crabs (*Portunus segnis*)	Blue crabs were obtained in fresh conditions. Shells were removed, washed, stored at -20 °C, macerated with solvent preparation (50:50 hexane:isopropanol) in a 30:1 solvent:raw material proportion under constant stirring (100 rpm, 120 h). Residual solvent was evaporated, and carotenoproteins were obtained with a petroleum ether:acetone:water (15:75:10 *v/v/v*) mixture (4 °C, 24 h).	The addition of carotenoproteins to sausages contributed to high inhibition zones of several gram negative (*E. coli*, *K. pneumoniae*, *S. enterica*, *Enterobacter* sp., and *S.* Typhimurium) and gram positive (*S. aureus*, *B. cereus*, *M. luteus*, and *E. faecalis*) bacteria, and fungi (*A. niger*, *F. oxysporum*, and *A. flavus*). Low TBARS (1.5–5.5 mg. MDA/kg sausages) and dienes formation; high heme iron (up to 6 μg/g sausages), and metmyoglobin contents (up to ~54%) were found in the manufactured sausages compared to control ones.	[130]
Cooked lamb patties	Alga (*Haematococcus pluvialis*)	Astaxanthins from *H. pluvialis* were part of a commercial dietary supplement containing 1% astaxanthin and excipients such as maltodextrin, magnesium stearate, and silicon dioxide. Astaxanthins (20, 40, and 60 ppm) were added to ground patties prepared from lamb legs prepared at 5 °C. For the cooked patties, antioxidants were added at 4 °C and left for 5 days, and then the patties were cooked in a convection oven (150 °C, 15 min) until the core reached 70 °C.	Astaxanthin-added patties displayed no differences (*p* > 0.05) in the pH levels (5.58–5.68) with control patties, but TBARS values were significantly lower (*p* < 0.05, −21.55 to −41.44%). The developed patties exhibited the same L* values, but higher a* and b*. The lowest TBARS levels were shown for the astaxanthin-patties, and cooked patties with astaxanthin displayed lower 7-α-hydroxycholesterol; 7-β-hydroxycholesterol; 5,6-β-epoxycholesterol; cholestan-3,5-dien-7-one, and 7-ketocholesterol than control patties.	[75]
Ground pork patties	Pitaya (*Hylocereus costaricensis*)	Pitaya peels were removed, air-dried (25 °C), milled (125 μm sieve), and stored in amber flasks. A microwave-assisted extraction was conducted by mixing 0.5 g of the powder with 25 mL ethanol (400 W, 30 s), followed by centrifugation (1400× *g*, 15 min, 4 °C), and supernatant collection. The resulting extract was concentrated by rotary evaporation (50 rpm, 60 °C under vacuum), maltodextrin was added, and the mixture was spray-dried (feed flow: 1 kg/h, air pressure: 7 bar; IAT: 170 °C, OAT: 90 °C). The extract was then vacuum packed and frozen (−80 °C). Two concentrations (100 and 1000 ppm) were added to pork patties prepared from pork loin (*Longissiums thoracis et lumborum*, 82% lean and 18% fat).	Formulated patties showed the lowest pH values (~5.5 to 6.0), higher L* (11.79–13.61%), and lower b* (−4.56 to −7.75%) along storage time (9 days). During the same shelf-life analysis, cohesiveness and springiness were preserved in the patties, but hardness and chewiness increased. Overall low TBARS (<3.5 mg MDA/kg meat) were obtained.	[132]
**Other food products**				
Pasta	Carrot (*Daucus carota* sbsp. sativus)	Minimally processed carrot residues (peel, shavings, and peduncles) were cleaned (chlorine solution: 200 ppm, 15 min), ground (125 μm), and added to pasta formulations (10–20% *w/w*).	Carrot flour mainly contained lutein (320.98 g/100 g), zeaxanthin (109.12 g/100 g), cryptoxanthin (143.75 mg/g), α-carotene (4296.78 g/100 g), β-carotene (4429.77 g/100 g), and retinol (340.75 g/100 g). Formulated carrot pasta showed higher solid loss (7.55–11.71%) and weight increase (216.27–220.49%), and significantly higher (*p* < 0.05) DPPH inhibition (21.02% vs. 10.01%) than control pasta.	[133]
	Saffron (*Crocus sativus*)	Saffron powder was commercially acquired and added (0.1, 0.2, and 0.4% *w/w*) to pasta (70% wheat flour and 30% water). Saffron dispersions were previously prepared with water and filtered (Whatman n° 40 paper).	Saffron-enriched pasta increased a* and b* values, decreased luminosity, and did not affect harness, cohesiveness, elasticity, nor chewiness, compared to control pasta. Saffron allowed high DPPH values (0.5–7.0-fold higher than control), and the formulated pasta was positively scored in terms of aspect, color, aroma, taste, and global acceptability.	[134]
Fruit jam	“Senduduk” fruit (*Melastoma malabathricum* L.)	Chopped “Senduduk” (purplish-black color) was blended with water (1:3 water:fruit proportion) and filtered with a gauze. Jackfruit (45 g) was mixed with sugar, 0.5 g citric acid, 1 g pectin, and the blend was boiled and stirred. After cooling (40 °C), and senduku extracts were added (2–10%). The product was cooked at 50 °C for 5 min until jam was formed.	Senduku provided vitamin C (2.81–3.02 ppm), increased pH along with concentration (3.4–3.7), and decreased total acidity from jackfruit jam. Moreover, senduku delivered b-carotene, ANC, TPC, and antioxidant capacity (IC_50_: 83.89–102.01 ppm).	[135]
Mayonnaise-like dressing sauce	*Muriellopsis* sp. alga	Lutein oleoresin was prepared from the freeze-dried biomass of *Muriellopsis* sp. at a final concentration of 20% *w/w*, prepared using vegetable oil. The solution was ultrasound-homogenized (40 oscillations, 6 pulses/s, 10 min).	Formulated mayonnaises exhibited higher lutein and pigment stability than commercial mayonnaises.	[136]

ΔE: Color difference against control samples, in the L*C*h* color space; a*: Redness color change; b*: Yellowness color change; ANC: Anthocyanins; C3G: Cyanidin-3-*O*-glucoside; cfu: colony forming units; cP: Centipoise; D3G: Delphinidin-3-*O*-glucoside; DM: Dry matter; DPPH: 2,2-diphenyl-1-picrylhydrazyl; IAT: Inlet air temperature; L*: Luminosity or Lightness; MDA: Malonaldehyde; OAT: Outlet air temperature; RSA: Radical scavenging activity (%); RSM: Response surface methodology; TAC: Total anthocyanin content; TBARS: Malonaldehyde acid reactive substances; TEAC: Trolox equivalent antioxidant capacity.

## Data Availability

Data are available upon reasonable request.

## Abbreviations

ΔE	Color difference against control samples, in the L*C*h* color space
a*	Redness color change in the CIEL*a*b* space
ABTS	2,2-azino-bis(ethylbenzothiazoline-6-sulfonic acid)
a_w_	Activity of water
ANC	Anthocyanins
b*	Yellowness color change in the CIEL*a*b* space
C*	Chroma
C3G	Cyanidin-3-*O*-glucoside
C3G-Mal	Cyanidin-3-(6′-malonylglucoside)
Caco-2	Human colorectal adenocarcinoma cells
cfu	Colony forming units
D3G	Delphinidin-3-*O*-glucoside
DM	Dry matter
DPPH	2,2-diphenyl-1-picrylhydrazyl
EC3G/L	Equivalents of C3G
EC_50_	Half-maximal effective concentration
EF	Electric field
EGCG	Epigallocatechin gallate
FRAP	Ferric ion reducing antioxidant power
FT-IR	Fourier Transform Infrared
GAE	Gallic acid equivalents
GC	Gas chromatography
GRAS	Generally recognized as safe
HepG2	Human hepatocellular carcinoma cells
HHPE	High hydrostatic pressure extraction
HPE	High-pressure-assisted extraction
IAT	Inlet air temperature
L*	Luminosity or lightness in the CIEL*a*b* space
MBC	Minimum bactericidal concentration
MCF-7	Human mammary gland/breast adenocarcinoma cells (derived from metastatic site)
MDA	Malonaldehyde
MDA-MB-231	Triple negative human mammary gland/breast adenocarcinoma cells
MIC	Minimum inhibitory concentration
OAT	Outlet air temperature
OH	Ohmic heating
P3G	Peonidin-3-(6′-malonylglucoside)
P3G-Mal	Pelargonidin-3-(6′-malonylglucoside)
PEF	Pulsed electric fields
Pr3G	Pelargonidin-3-*O*-glucoside
RSA	Radical scavenging activity (%)
SC-CO_2_	Supercritical carbon dioxide
SFC	Supercritical fluid chromatography
SFE	Supercritical fluid extraction
TAC	Total anthocyanin content
TBARS	Thiobarbituric acid reactive substances
TEAC	Trolox equivalent antioxidant capacity
TPC	Total phenolic compounds
VPR	Vine-pruning residues
XR	X-ray
